# A Review on Organic Photosensitizers for Hydrogen Evolution by Water Splitting

**DOI:** 10.1021/acsomega.6c01074

**Published:** 2026-04-13

**Authors:** Lucia Ivanová, Jan Truksa, Kyusun Kim, Dong Ryeol Whang, Bong Sup Shim, Jozef Krajčovič

**Affiliations:** † Institute of Chemistry and Technology of Environmental Protection, Faculty of Chemistry, Brno University of Technology, Purkyňova 118, Brno CZ-612 00, Czech Republic; ‡ Center for Clean Technology, 26718 Inha University, 100, Inha-ro, Michuhol-gu, Incheon 22212, Republic of Korea; § Department of Chemical Engineering and Program in Biomedical Science and Engineering, 26718 Inha University, 100, Inha-ro, Michuhol-gu, Incheon 22212, Republic of Korea; ∥ Department of Advanced Materials, Hannam University, Daejeon 34054, Republic of Korea

## Abstract

Hydrogen is increasingly recognized as a key energy vector for future low-carbon energy technologies. Solar-driven water splitting offers a direct and sustainable route to its production, with organic photosensitizers (OPS) emerging as a tunable and synthetically accessible alternative to traditional semiconductors. This review summarizes recent progress in OPS for photocatalytic hydrogen evolution, encompassing diverse molecular architectures ranging from noble-metal-based complexes to noble-metal-free analogues and fully metal-free organic dyes. Particular emphasis is placed on sustainable materials design, including the use of earth-abundant elements and modular synthetic strategies. We further examine the integration of OPS into polymeric and hybrid material systems—such as metal–organic frameworks (MOFs), conjugated polymers (CPs), and polydopamine (PDA)—that enhance structural stability and light-harvesting performance. Key design principles, charge transfer dynamics, and the influence of cocatalysts and sacrificial reagents are discussed. The review concludes by outlining challenges and future directions for developing efficient, stable, and scalable OPS-based platforms for solar hydrogen production.

## Graphical abstract

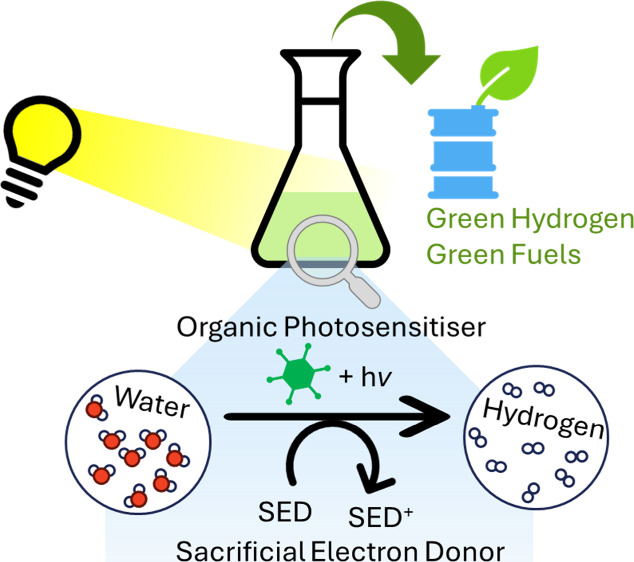

## 1 Global Perspective

As the world navigates increasingly complex energy and environmental challenges, the urgency for sustainable and long-term solutions has never been greater. The continuous overexploitation of fossil fuels has not only led to resource depletion but also significantly intensified global warming (Figure 1). Although international frameworks such as the Kyoto Protocol and the Paris Agreement have helped define climate goals, carbon emissions remain on the rise, and the target of climate neutrality becomes progressively more difficult to achieve.^1,2^ If current trends persist, then a critical threshold, commonly referred to as the “point of no return”, may be crossed within the next decade. This situation places a growing responsibility on the scientific community to explore scalable and clean technologies. Among them, the generation of hydrogen using solar energy stands out as a particularly promising route toward sustainable fuel production.

**Figure 1:**
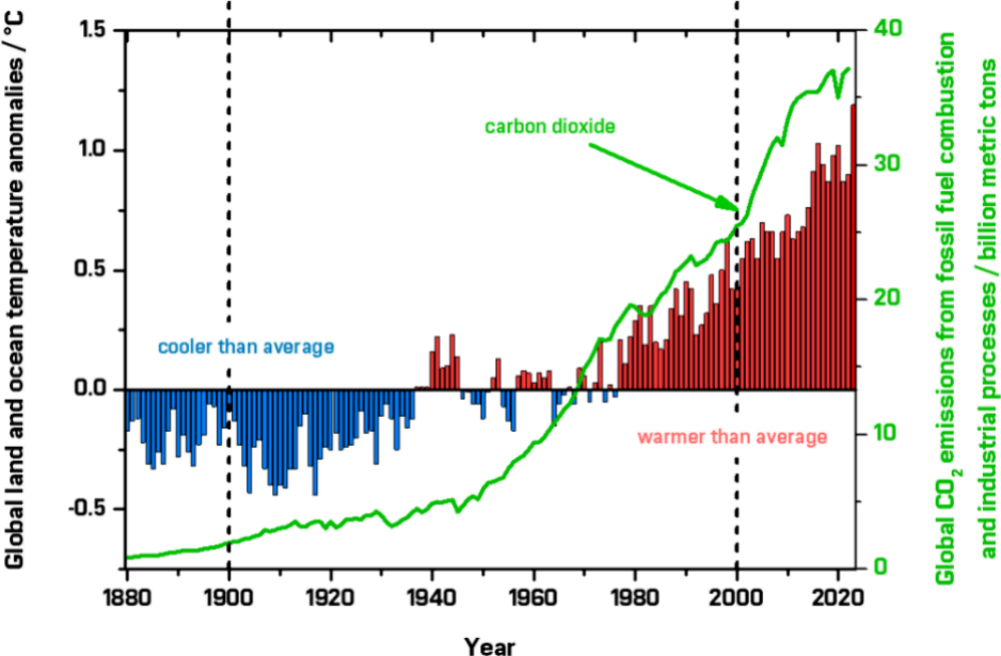
Notable correlation between annual anomalies in global and land surface temperatures (based on temperature departure/°C; red—positive deviation, blue—negative deviation, compared to mid-20th century average)^3^ with annual global CO_2_ industrial and combustion emissions.^4^ Data were retrieved from refs 5,6 Reference provided for context; not reproduced.

Hydrogen offers the highest gravimetric energy density of all practical fuels (142 MJ·kg^–1^), significantly exceeding traditionally used hydrocarbons such as methane (56 MJ·kg^–1^), gasoline (47 MJ·kg^–1^), and diesel (45 MJ·kg^–1^).^7,8^ At present, it is the most widely used industrial gas, serving essential roles in ammonia synthesis via the Haber–Bosch process, methanol production, hydrogenation of unsaturated fats in food processing, petroleum refining, metallurgy, and as a cryogenic propellant in aerospace applications,^9,10^ with applications in ammonia and methanol production, petroleum refining, metallurgy, and food processing.^11^ In parallel, its clean combustion and versatility across sectors position hydrogen as a key vector in the transition to carbon-neutral economies.

Despite its high potential, more than 90% of global hydrogen is still produced from fossil-based feedstocks, predominantly through steam methane reforming, which is energy-intensive and releases substantial CO_2_.^12–14^ To enable a meaningful transition to green hydrogen, countries have launched dedicated national strategies. Japan was among the first with its Basic Hydrogen Strategy (2017),^15^ followed by the European Union’s Hydrogen Roadmap (2020).^16^ More recently, South Korea, Australia, China, and the United States have also adopted national frameworks to support hydrogen innovation across sectors, including transport, industry, and grid-scale storage.^17–23^

Photocatalytic water splitting is one of the most direct and energy-efficient approaches to green hydrogen production, a concept with roots in early 20th-century scientific vision. As early as 1912, Giacomo Ciamician proposed the idea of harnessing sunlight to drive chemical reactions, anticipating the principles of artificial photosynthesis.^24^ Decades later, the groundbreaking work of Honda and Fujishima in 1972 demonstrated the photocatalytic splitting of water on TiO_2_ under ultraviolet light, providing a practical foundation for solar hydrogen generation.^25,26^ This method utilizes solar energy and avoids the need for a high external electrical input, making it particularly suitable for decentralized or off-grid applications. The development of stable and efficient photocatalytic systems could reduce the reliance on central infrastructure and enable on-site hydrogen generation for a range of uses.

However, technological feasibility alone does not ensure widespread adoption. Public trust in hydrogen safety remains a limiting factor. Hydrogen is often perceived as inherently hazardous due to its high flammability and use in high-pressure systems. Nonetheless, it is nontoxic, lighter than air, and disperses rapidly in the event of a leak—making it less prone to accumulation and ignition compared to heavier fuels like gasoline or natural gas.^27,28^ With proper engineering controls, modern materials, and appropriate safety measures, hydrogen can be handled as safely as any other industrial fuel.

Storage and transport still pose technical challenges, particularly due to hydrogen’s low volumetric density and the risk of material embrittlement. Current solutions include physical storage such as high-pressure composite tanks, cryogenic storage, geological storage,^11,29^ and chemical carriers such as ammonia^30–32^ or hydrides.^27^ An emerging alternative is the integration of in situ hydrogen production with proton-exchange membrane fuel cells, which create compact, self-sustained energy systems.

In this context, organic photosensitizers (OPS) are especially promising. Composed of earth-abundant elements, they offer tunable photophysical properties, control over redox potentials, and compatibility with diverse light-harvesting and catalytic systems. Their processability and potential for hybrid integration also make them suitable for flexible, scalable platforms. Continued research into OPS-based photocatalytic systems directly contributes to the broader goals of clean hydrogen generation, energy decentralization, and long-term environmental resilience.

## 2 Introduction

The rising global demand for sustainable energy has intensified efforts to develop clean technologies for fuel production. Among these, hydrogen stands out as a promising energy carrier, offering a high gravimetric energy density and producing only water upon combustion. Photocatalytic hydrogen evolution has emerged as a viable strategy for harnessing solar energy to generate hydrogen from water—a process that aligns with the goals of carbon-neutral energy systems.^33–36^

Currently, several methods are employed to produce hydrogen, including water electrolysis,^37–39^ photoelectrochemical water splitting,^40–42^ steam reforming of hydrocarbons,^43,44^ and hydrolysis of reactive metals and hydrides.^45,46^ Among them, photoelectrochemical and photocatalytic routes are considered more sustainable as they directly utilize sunlight to drive hydrogen generation. Central to these systems are photosensitizers (PS), molecular components that absorb light and mediate electron transfer required for the hydrogen evolution reaction (HER). The design of efficient PS is crucial as they significantly influence the quantum efficiency and overall stability of the photocatalytic system.

Inorganic semiconductors have long dominated photocatalytic water splitting due to their high photoelectronic conversion efficiency and structural robustness.^47^ For example, aluminum-doped SrTiO_3_ has demonstrated an external quantum efficiency of 96% under ultraviolet light (350–360 nm).^48^ However, because ultraviolet light constitutes less than 5% of the solar spectrum, visible-light-active photocatalysts are crucial to achieving the solar-to-hydrogen (η_STH_) conversion efficiencies (typically 5–10% needed) for commercial applications.^49,50^ This necessity has sparked interest in developing organic semiconductor-based photocatalysts. Although the HER values of organic photocatalysts in half-reactions are now comparable to those of their inorganic counterparts, challenges remain in terms of overall water splitting (OWS) efficiency and long-term stability.

In this context, molecular organic photosensitizers (MOPs) are gaining increasing attention. These systems, including small molecules and conjugated polymers, offer several advantages: they are composed of earth-abundant elements, exhibit tunable optical and redox properties through chemical modification, and are well-suited for integration into hybrid systems.^51^ Moreover, their compatibility with solution processing and flexible supports makes them appealing for scalable and device-oriented applications. Furthermore, organic systems are generally more amenable to the fabrication of hybrid materials, which can further enhance their performance by improving the light absorption, charge separation, and catalytic efficiency.

Despite these advantages, the development of MOP-based systems faces key challenges such as limited photostability, inefficient charge separation, and difficulty achieving sustained hydrogen evolution. Hence, designing stable OPS with high efficiency, broad light absorption, and effective electron transfer properties remains a critical objective in the field.^52,53^

This review provides a comprehensive overview of the recent advancements in MOPS for hydrogen evolution briefly summarized in Figure 2. It outlines key molecular design strategies, performance metrics, and structure–activity relationships. In addition, we highlight advances in polymeric photosensitizers, the integration of hybrid platforms including polydopamine (PDA) and metal–organic frameworks (MOFs), and the distinction between homogeneous and heterogeneous system architectures. Finally, we discuss current limitations and outline future directions toward efficient, stable, and scalable molecular photosensitizer systems for solar hydrogen production.

**Figure 2:**
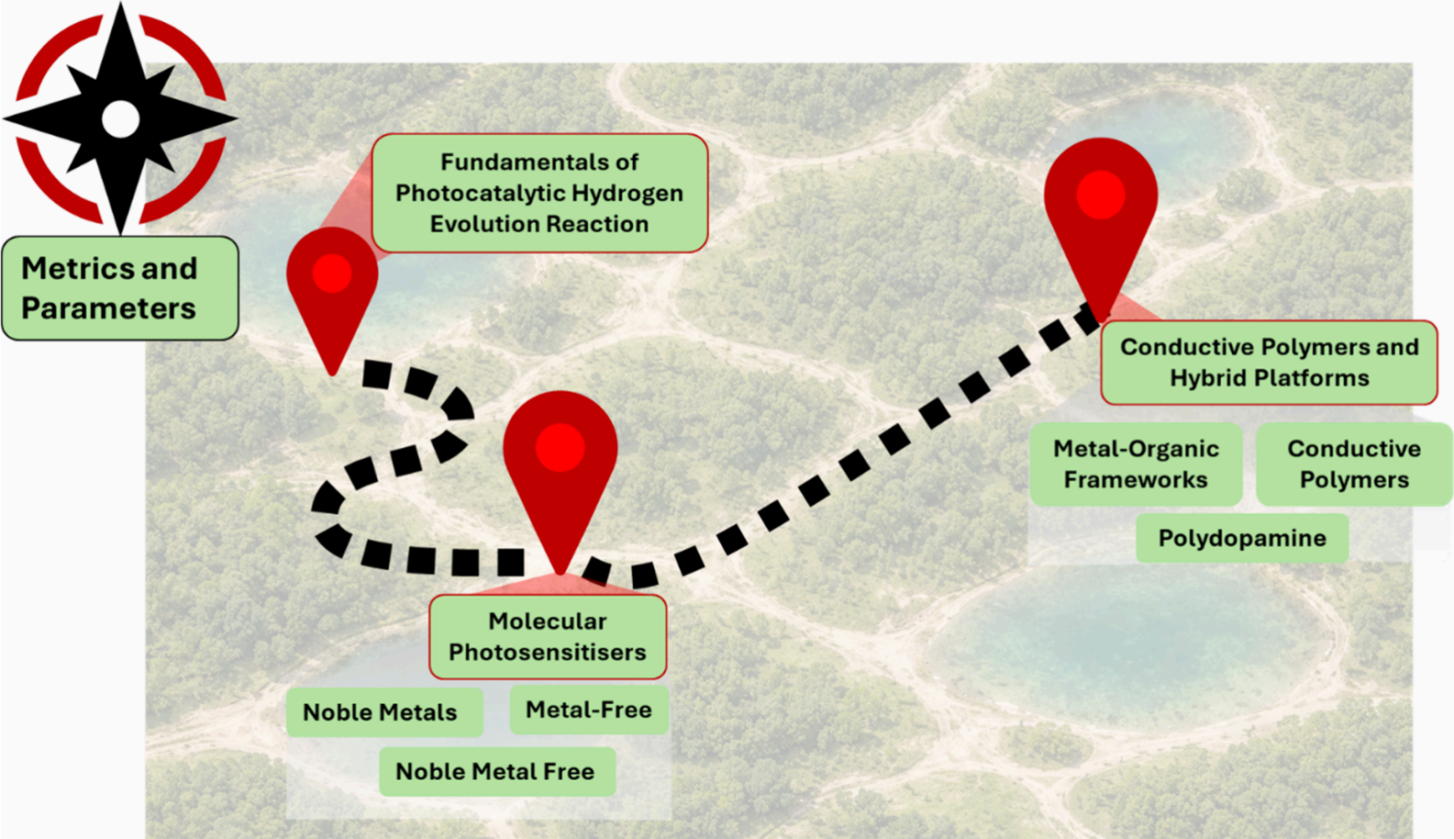
Simplified roadmap of the review—from the definition of metrics and parameters, through explaining the fundamentals of photocatalytic hydrogen production, across and overview of catalytic systems.

## 3 Performance Metrics and System Parameters

In this section, the most important factors needed to describe the system quantitatively will be mentioned. Plenty of techniques are used to describe the structure of the PS molecules and the various transition states during photoexcitation and charge transfer. These range from conventional NMR or UV/vis spectroscopy to transient Raman or X-ray analysis, electron paramagnetic resonance, or microscopy for heterogeneous systems. The great variety of instrumentation and experimental considerations provides enough material for its own review. Since an exhaustive and relatively recent paper has been published by Kranz and Wächtler^54^ we offer only a much shorter summary of the most impactful indicators.

### 3.1 Light Parameters and Spectral Considerations

The performance and efficiency of photosynthetic processes are heavily dependent on the light source, its light intensity, and the spectral profile of the emission wavelengths. Natural photosynthesis occurs upon “photosynthetically active radiation”, which closely matches the visible (VIS) part of the solar spectrum, ranging from 400 to 700 nm. At the same time, photons with shorter wavelengths, such as ultraviolet light (UV) and below, are too energetic and can potentially damage tissues and cells of the photosynthesizing organisms. Conversely, photons with longer wavelengths, such as near-infrared (NIR) light and above, do not carry enough energy to trigger photosynthesis effectively.^55^

In artificial photosynthesis, the choice of the light source is critical and must be tailored to the absorption properties of the photosensitizer. Reactors driven by sunlight are considered ideal for this purpose as the Sun is an abundant and renewable energy source, although the solar spectrum consists of only about 5% UV, 40% VIS, and 55% NIR.^56^ This uneven distribution of wavelengths presents challenges in fully utilizing the solar spectrum for efficient hydrogen production.^57,58^

So far, the majority of the laboratory-scale studies rely on synthetic light sources, including near-UV lamps,^59^ xenon arc lamps,^60^ halogen lamps, mercury lamps,^61^ solar simulators AM1.5 G (the global standard spectrum Air Mass 1.5 Global with total irradiance of 100 mW·cm^–2^),^62^ or light-emitting diode (LED) lights,^63^ matching the absorption peak of the used photosensitizers. Xenon arc lamps with continuous spectrum are often preferred for sunlight simulation in laboratory settings, while LED lamps offer high energy efficiency and tunability to a specific wavelength, allowing for targeted excitation of PS.

Lamps differ in spectral range, power, light intensity, or incident photon fluxes, causing difficulties in unifying hypothetical standards between laboratories. Moreover, xenon, mercury, and AM1.5 lamps have strong thermal effects that, if needed, must be suppressed by employing filters (e.g., water). Furthermore, the configuration of the light source and reactor strongly influences the performance, depending on the irradiated area, the distance that photons must overcome, and the construction material of the reactor. Therefore, for repeatability, it is necessary to mention all the critical and accurate characteristics when reporting the results.^64^

### 3.2 Hydrogen Detection Techniques

Gas chromatography (GC) remains a widely used and reliable laboratory technique for measuring a broad range of H_2_ concentrations. This method typically employs the thermal conductivity detector (TCD), which operates on the principle of detecting differences in thermal conductivity between the pure carrier gas (CG) and the target gas component (H_2_) dissolved in the CG. The advantages of using GC-TCD are the excellent resolution and separation power to distinguish H_2_ from other gas components, high sensitivity, relatively fast analysis time, and easy recording and evaluation of the data. However, the main drawback is the high initial cost of the instrument.^65,66^

The choice of CG, typically helium or nitrogen, can significantly impact the detector sensitivity due to the differences in thermal conductivities. For analysis of higher H_2_ concentrations in the samples, helium may cause nonlinearity and low response due to minor differences in thermal conductivity between helium and H_2_. In addition, helium is expensive and in short supply on Earth, primarily produced from uranium decay or as a byproduct of natural gas refinement. Often, it is considered a “non-renewable element” because it cannot be artificially produced.^67,68^

Conversely, nitrogen has a thermal conductivity lower than that of helium, which results in a more linear response and higher sensitivity in a broader range of H_2_ concentrations than helium. It is a popular, suitable, and more affordable option for CG. It is well-known for its inertness and abundance and is obtainable in the high purities required for accurate hydrogen analysis. The use of high-purity nitrogen helps to minimize the impact of impurities on the measurement, further enhancing the reliability of the analysis.^65^

The advanced analytic method uses the dielectric-barrier discharge ionization detector (BID) with higher sensitivity than TCD-detecting gas components at the ppm level.^69^ The principle lies in generating a stable helium plasma using a dielectric barrier discharge system to ionize separated compounds from the GC column. The ions are attracted to a collector and are detected as peaks. Compared to GC-TCD, GC-BID provides a higher sensitivity and reproducibility in H_2_ analysis. On the other hand, it uses helium as a CG, which is the main drawback of this method due to the reasons stated above.^70,71^

### 3.3 Efficiency Metrics and Benchmarking

As mentioned previously, the efficient absorption of light is a necessary condition for artificial photosynthesis. Furthermore, since the photocatalytic system should utilize solar light, the absorption of the photosensitizer molecule should be tuned to the 500–600 nm spectral band, where sunlight has the highest spectral irradiance.^72^ Fortunately, organic synthesis allows for fairly straightforward tuning of the Frontier Molecular Orbital (FMO) energies, which have a decisive influence on the absorption of electromagnetic waves.^73^ In addition, computational chemistry has become an important tool in solar-fuels research, not only for predicting optoelectronic properties, but also for elucidating catalytic mechanisms and guiding the rational design of bioinspired systems.^74^ Further, today’s technology makes it possible to predict the absorption bands of organic molecules via Density Functional Theory (DFT) methods. While the exact explanation of DFT and a survey of common methodologies are outside the scope of this review, we can direct the reader to several excellent sources.^75–78^ An older but still relevant paper is the benchmark of Time-dependent DFT methods performed by Jacquemin et al.^79^

#### 3.3.1 Material-Based Figures of Merit

When a molecular system is used, a careful selection of figures of merit is required to evaluate the light-driven HER performance. The quantitative assessment of the system’s efficiency and stability are crucial factors for determining the process viability.

H_2_ production is commonly determined by the popular metrics turnover number (TON) and turnover frequency (TOF), which completely neglect the influence of light. Further, the TON is dependent on the time of measurement (Table 1). The TON describes the stability and durability of the system during its lifetime before it becomes deactivated, while TOF measures the instantaneous efficiency and the intrinsic activity of the system.^80,81^ It is important to note that the reaction conditions, such as the light source, temperature, reaction time, reaction medium, or type of reactor, influence the TON and TOF values. Thus, TON and TOF can be reliably used to compare the performance of identical or very similar molecular catalyst systems. However, it is not recommended to directly compare the values between fundamentally different systems, as the differences may be attributed to the varying experimental conditions rather than the intrinsic properties of the catalysts.^64,82^

**Table 1:** Material-Based Figures of Merit

**turnover number** (TON) dimensionless quantity	turnover frequency (TOF) unit: reciprocal of time
the number of moles of H_2_ molecules *n*_*H*2_ produced per moles of photocatalyst/cocatalyst molecules in the ground state *n*_cat_,^83,84^ *Z* is the number of electrons involved in the process (for H_2_ molecules, *Z* = 2)	the number of H_2_ molecules produced per mole of photocatalyst/cocatalyst molecules in the ground state per unit of time^83,84^
$\mathrm{TON}=\frac{Z\times n_{H_{2}}}{n_{\mathrm{cat}}}$	$\mathrm{TOF}=\frac{Z\times n_{H_{2}}}{n_{\mathrm{cat}}}\times \frac{1}{t}$ $\mathrm{TOF}=\frac{\mathrm{TON}}{t}$

#### 3.3.2 Light-Based Figures of Merit

In addition to the material-based TON and TOF, light-based figures of merit provide insights into the efficiency of light energy conversion to products, however, neglecting the concentration of photosensitizer or cocatalyst (Table 2).

**Table 2:** Light-Based Figures of Merit

**quantum yield** (QY)	**apparent quantum yield** (AQY)	**solar-to-hydrogen conversion efficiency** **(η**_**STH**_ **)**
the ratio of the number of electrons participating in the reaction per photon of light of a specific wavelength that is absorbed by the photosensitizer/photocatalyst^83–85^	the ratio of the number of electrons involved in a specific photocatalytic reaction to the number of photons absorbed by the photocatalyst, where *Z* is the number of electrons, $r_{H_{2}}$ is the rate of hydrogen production. The number of absorbed photons is calculated based on the derivation of Kisch and Bahnemann^86^ or as the difference of the incident and transmitted photon flux, in case the experimental setup is able to measure both^87,86^	the energy conversion efficiency from input solar energy to producedchemical energy, where $r_{H_{2}}$ –the rate of H_2_ production, Δ*G*–gain in Gibbs energy (237 kJ·mol^–1^), *P*_sun_–sunlight energy flux, *S*–the effective area of the reactor^64,88^
$\phi \left[\%\right]=100\times \frac{N_{\mathrm{reacted}\;\mathrm{electrons}}}{N_{\mathrm{absorbed}\;\mathrm{photons},\lambda }}$	$\mathrm{AQY}\left[\%\right]=100\times \frac{Z\times r_{H_{2}}}{N_{\mathrm{absorbed}\;\mathrm{photons}}}$	$\eta_{\mathrm{STH}}\left[\%\right]=\frac{\mathrm{chemical}\;\mathrm{energy}\;\mathrm{output}}{\mathrm{solar}\;\mathrm{energy}\;\mathrm{input}}\times 100\%$ $=\frac{r_{H_{2}}\left[\mathrm{mmol}\cdot s^{-1}\right]\times \Delta G\left[\mathrm{kJ}\cdot {\mathrm{mol}}^{-1}\right]}{P_{\mathrm{sun}}\left[\mathrm{MW}\cdot {\mathrm{cm}}^{-2}\right]\times S\left[{\mathrm{cm}}^{2}\right]}\times 100\%$

The determination of the quantum yield (QY) requires irradiating the system with monochromatic light while considering any light losses due to the reactor setup.^85^ However, this is experimentally more difficult to achieve and is not realistic for a real-world application efficiency determination. Therefore, in a similar context, the apparent quantum yield (AQY) is used as an indicator of photocatalytic activity. Assuming the solution is perfectly homogeneous, and the absorbance of the solution is very small (below 0.05), the *I*_a_ can be determined as the product of the incident photon flux, the molar absorption coefficient of the photosensitizer, and the optical length of the reactor.^87,86^ It should be noted that AQY is defined for photons of a single wavelength (energy). In order to describe multiwavelength light, it is necessary to determine AQY values across the spectrum or to use the analogically defined Apparent Quantum Efficiency (AQE) with integral photon flux values to cover multiple wavelengths.

The AQY has a decisive influence on the rate constant of the reaction, which makes the reaction rate dependent on the spectral composition and intensity of the incident light, and construction of the reactor (especially optical length), as well as the PS and substrate concentration - for a more detailed discussion of this phenomenon, including a derivation of the rate constant formula, the reader is referred to the paper of Kisch and Bahnemann.^86^ For this reason, the reaction rate is heavily dependent on the experimental setup, and spectrally resolved AQY values seem to be a much better tool for comparing different photocatalytic systems.

As mentioned above, the overall efficiency of the H_2_ reduction is dependent on the width of the PS absorption band. The efficiency in lower-wavelength spectral bands is limited by a relatively low number of photons, and the efficiency in higher-wavelength spectral bands is limited by the lower energy of the photons. Therefore, a fairly specific window of quantum yields and absorption characteristics exists where the overall η_STH_ reaches appreciable levels. Briefly, the AQY of a successful system must be above 40% in the spectral band of approximately 500–700 nm. Such a system will have an overall η_STH_ of over 10%, which should be commercially viable. For a more detailed discussion, the reader is referred to older but still valuable works by Hisatomi et al.^87^ and Pinaud et al.^89^ While the latter is focused on heterogeneous catalysis, it still contains a useful economic analysis of the process on an industrial scale.

Finally, η_STH_ is a critical performance metric for photoelectrochemical (PEC) water-splitting systems, and recently, it was accepted as a standard measure for artificial photosynthesis and light-driven water-splitting.^90^ It indicates the practical application potential under solar irradiation and reflects the conversion efficiency of incident solar energy into hydrogen via the water-splitting reaction by a photocatalyst. Notably, according to the definition, it applies only to the AM1.5G spectrum and requires information about the reactor geometry. Further, the η_STH_ has an intrinsic relationship to the absorption spectrum of the photosensitizer; briefly, there are many fewer UV and photons in the blue spectral band than, e.g., in green or yellow bands of solar radiation incident on Earth’s surface. Therefore, a photosensitizer absorbing below 400 nm can only reach around 1.7% η_STH_ since it is unable to absorb most of the incident photons.^87,91,92^ On the other hand, the definition neglects the photocatalyst’s concentration and number of active sites. This allows the η_STH_ metric to be used reliably for homogeneous and heterogeneous light-driven water-splitting systems.^85,87,88,93–95^

Further, it is necessary to test the stability of a photocatalytic system, generally by carrying out the reaction over many hours and checking for signs of photodegradation, such as a lowered absorbance or hydrogen production rate. In this context, various *in operando* techniques prove highly useful.^96^ Indeed, the possibility of carrying out FTIR, NMR, or X-ray absorption spectroscopy measurements with millisecond resolution may prove decisive in investigating both photocatalytic mechanisms and degradation processes and open the door to a new generation of tailor-made molecules for specific applications^97^

Finally, we mention the economic and environmental costs of the catalytic system as a decisive factor. To minimize both, natural and nontoxic precursors are recommended. In this context, carbazole-based copper complexes^98^ and methylene blue^99^ can be mentioned. The search for similar simple and cost-efficient synthetic pathways to new photosensitizers remains a key element of research. Further, the synthesis should have as few steps as possible and utilize a benign solvent, such as water or ethanol. In this regard, we may direct the reader to a recent review focused on the toxicological aspects of water splitting by Khalkhali et al.^100^

## 4 Fundamentals of Photocatalytic Hydrogen Evolution

Natural photosynthesis, arguably considered “the most important life-maintaining process on Earth”, uses solar energy to drive the otherwise energetically unfavorable transformation of carbon dioxide and water into oxygen and a higher energy compound, glucose.^101^ Detailed mechanistic studies of Photosystem II, particularly its water-oxidation cycle and the associated redox transitions of the oxygen-evolving complex, have provided a key conceptual framework for the design of artificial solar-fuel systems.^102,103^ In contrast, artificial photosynthesis aims to mimic the natural process and utilize it to produce strategic and economically valuable substances like molecular hydrogen, methane, or methanol. So, the energy of the largest, could-be-claimed infinite, available renewable energy source, the Sun, is stored in chemical bonds.^104^

### 4.1 From Photocatalysis to Photosynthesis: Defining the Scope

Light-harvesting systems utilize the energy of the absorbed light to drive chemical conversions that are either difficult or even impossible to carry out in the dark. Generally, these systems are classified as “excitonic chemical conversion systems” as they promote the chemical reaction *via* an excited state. The literature commonly refers to the concept as “photocatalysis”, “photosynthesis”, or “artificial photosynthesis”; even though the terms are very likely interchangeable in the scientific community, the difference between the terms needs to be clarified.^105,106^

The definition of the term “photocatalyst”, according to the International Union of Pure and Applied Chemistry (IUPAC), is “*a catalyst able to produce, upon absorption of light, chemical transformation of the reaction partners. The excited state of the photocatalyst repeatedly interacts with the reaction partners, forming reaction intermediates and regenerating itself after each cycle of such interactions*”.^107^ This refers to all excitonic chemical conversion applications. Therefore, a more specified description by Bard distinguishes the nuance in terms of the thermodynamics of the particular process.^108^

Photocatalysts use light to accelerate chemical transformations that are thermodynamically downhill (Δ*G* < 0). There is no change in the thermodynamics but only the kinetics of the reaction. On the other hand, photosynthetic systems, including both natural and artificial, drive thermodynamically uphill (or forbidden) reactions (Δ*G* > 0), and require light energy input for the suppression of the favorable reverse reaction.^83,106^

Thus, when performing light-driven redox reactions, the Δ*G* sum of both half-reactions determines whether the process is photocatalytic or photosynthetic. Referring to water splitting, it is justified to use the term “photosynthesis” (Δ*G* = +241.3 kJ·mol^**–**1^). However, the use of various sacrificial reagents, e.g., methanol or glycerol (as known as sacrificial water splitting), in conjunction with the hydrogen evolution half-reaction can often result in an energetically downhill process, photocatalytic in nature.

### 4.2 Mechanistic Principles of Photocatalytic Hydrogen Evolution

In inorganic-based photocatalytic hydrogen production, metal oxide semiconductors convert light energy into chemical energy through redox reactions, with their electronic structure playing a critical role in determining catalytic efficiency.^109,110^ Unlike conductors, semiconductors possess distinct valence bands (VB) and conduction bands (CB), with the energy difference between these bands defined as the band gap (*E*_g_). In the ground state, electrons and holes are localized within the VB. Upon photon absorption, when the photon energy meets or exceeds the band gap, electrons are excited from the VB to the CB, resulting in the formation of holes. These photoexcited electrons and holes can recombine either within the bulk of the material or at the surface, releasing energy in the form of heat and light.^111^ Alternatively, those electrons and holes that reach the surface without recombining can participate in redox reactions—where electrons drive reduction processes, and holes facilitate oxidation processes. These surface reactions are fundamental to the mechanism of photocatalytic hydrogen production utilizing metal oxides (Figure 3a).^112^ Despite the potential of metal-oxide-based photocatalytic water-splitting, the energy conversion efficiency remains insufficient for practical hydrogen production. To overcome this limitation, the integration of PS has emerged as a promising strategy to enhance photocatalytic performance, particularly by broadening the absorption spectrum into the visible light range. PS significantly improves the efficiency of solar-to-hydrogen conversion by extending the absorption capabilities of metal oxides, which typically exhibit limited performance in this spectrum. Upon irradiation with visible light, the excited PS can inject electrons into the conduction band of the semiconductors, thereby enhancing the overall photocatalytic activity. This expanded light absorption not only increases the utilization of solar energy but also accelerates electron transfer, maintaining continuous catalytic activity (Figure 3b).^113–115^ Consequently, the integration of PS and semiconductors facilitates more efficient photon-to-hydrogen conversion, rendering this approach highly promising for practical solar-driven hydrogen production systems. Moreover, the efficacy of these PS in hydrogen generation is significantly influenced by their operational environment, which can be broadly categorized into homogeneous and heterogeneous systems. Understanding these environments is critical as they affect the performance, stability, and overall efficiency of the photocatalytic processes involved in hydrogen generation. In heterogeneous systems, solid-state architectures are employed, wherein organic PS are immobilized on surfaces or embedded within porous materials. (Figure 3c).^110,116^

**Figure 3:**
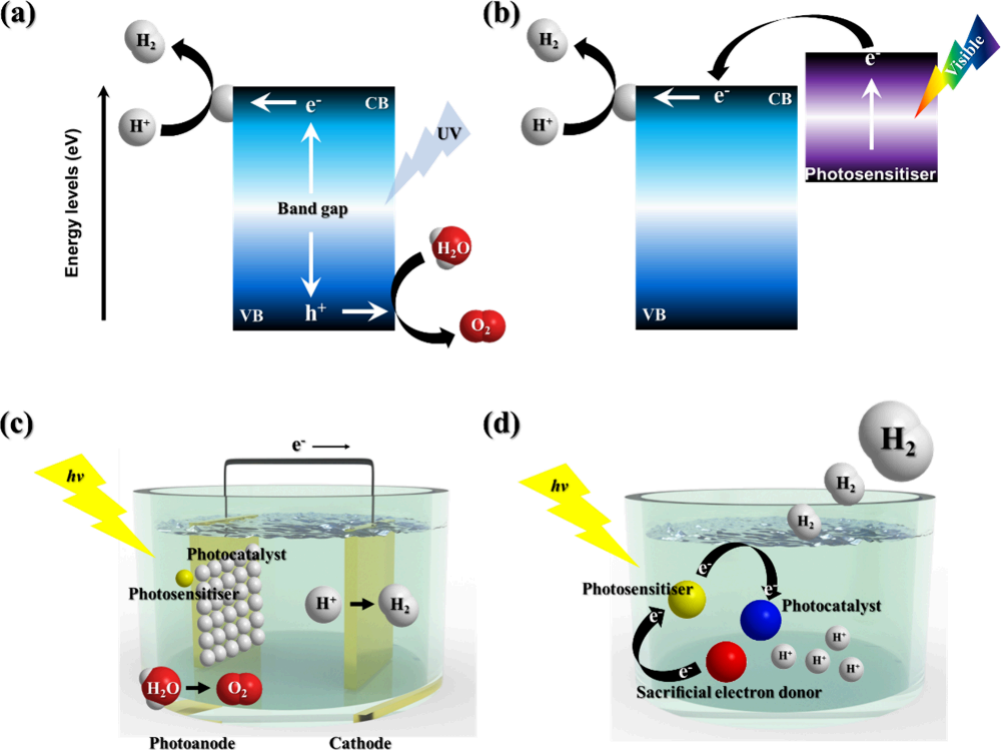
Illustration of (a) the hydrogen evolution mechanism using a metal oxide system as a photocatalyst, (b) the hydrogen evolution mechanism using a PS, (c) a heterogeneous system, and (d) a homogeneous system in water-splitting hydrogen evolution.

This immobilization enhances stability, reduces electron–hole recombination, and improves recyclability, making these systems more suitable for long-term hydrogen production. However, heterogeneous systems face challenges related to efficient electron transfer across phases and optimization of mass transport within solid–liquid interfaces. Conversely, homogeneous systems present distinct advantages that mitigate some of these challenges.

For example, the intimate molecular interactions in homogeneous systems allow for the coexistence of all components (PS, catalysts, and electron donors) within the same phase, thereby facilitating high energy transfer efficiency. (Figure 3d).^117^ Since the role of catalysts and electron donors is crucial, we offer a brief discussion of the most commonly used systems in the Supporting Information (SI) section.

This proximity enhances the likelihood of exciton diffusion and charge separation, leading to an improved photocatalytic performance. Furthermore, researchers can customize and tune organic PS for specific wavelengths of light, enabling the design of synergistic combinations that can enhance the hydrogen production rates. Additionally, the single-phase nature of homogeneous systems simplifies the investigation of reaction mechanisms, allowing for a more straightforward monitoring of kinetics and electron transfer processes. This results in high reaction rates due to rapid charge transfer among redox-active species. Moreover, the precise control over reaction conditions—such as concentration, pH, and temperature—enables optimization of hydrogen production. Homogeneous systems can also be readily integrated with advanced photochemical techniques and adapted for scalable processes, whether in batch or continuous flow formats.^118–120^

Since this review is primarily focused on organic photosensitizers, we offer a more detailed mechanism of a general H_2_-evolving system employing MOPS, and the energetic requirements for the HOMO/LUMO orbitals of organic compounds or CB and VB of inorganic semiconductors, respectively, are illustrated in Figure 4 for the HER half reaction and OWS cases. While OWS without sacrificial reagents is essential to producing green hydrogen cost-effectively and achieving commercialization. However, it is still a challenging task due to the complicated charge interplay involving light absorption, charge separation, transfer, and collection. Further, the optimal environments, i.e., pH, solvent, for HER and OER often differ significantly, and OER, a 4-electron process, is generally slower than HER, a 2-electron process, thus limiting the overall reaction kinetics of the entire system.

**Figure 4:**
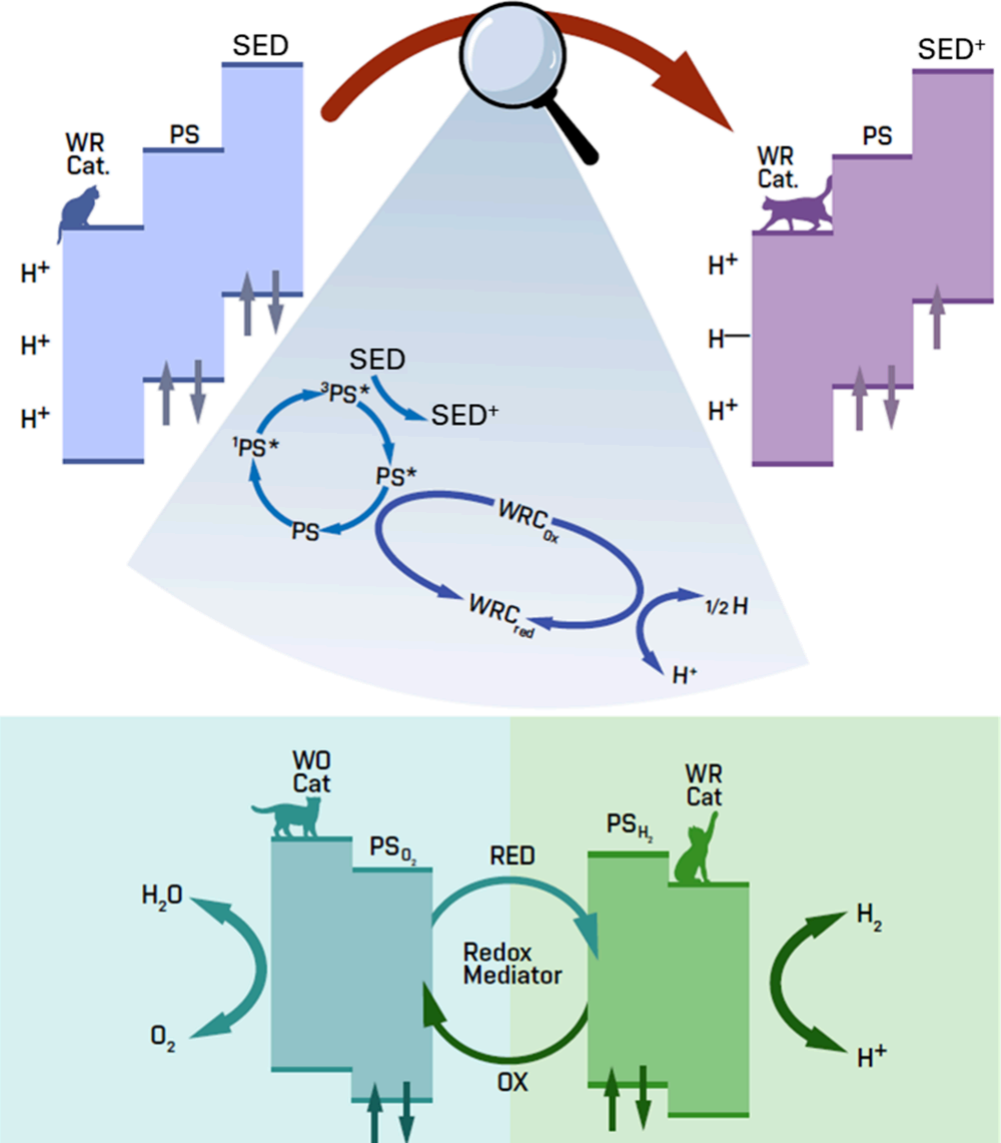
Two possible pathways for HER: Top–Schema of the water-reduction mechanism (via reductive quenching) employing sacrificial electron donor (SED); Bottom General illustration of the Z-scheme catalysis for overall water splitting–hydrogen reduction and water oxidation with the presence of a redox mediator. PS–photosensitizer, WR Cat (WRC)–water reduction catalyst, WO Cat (WOC)–water oxidation catalyst.

Due to these challenges, researchers have primarily focused on optimizing each reaction independently through half-reactions using sacrificial reagents.^121^ Although there have been attempts to circumvent this issue, they are unfortunately still plagued by low efficiency and stability. To name a few examples, the porphyrin-based Z-scheme system of Wang et. al.^122^ and the spin-hybrid semiconductors of Lin et al. have apparent quantum yields (AQY—See Chapter 3) below 10%. Another possibility is dye-sensitized photoelectrochemical cells, which are complicated, generally unstable, and require external electrical bias to reach high efficiency.^123–125^ Finally, while poly (triazine imide) crystals reach AQY above 10%, they require UV light to work, thus utilizing only a small part of the solar spectrum, and the OER half-reaction relies on a cobalt oxide cocatalyst, introducing a toxic heavy metal.^126,127^

In the following sections, we will examine various PS in detail, emphasizing their roles and contributions to optimizing hydrogen production. Additionally, we discuss the enhanced hydrogen production achieved through the integration of PS with metal–organic frameworks, conducting polymers, and polydopamine, the latter of which is recognized as an environmentally friendly and biocompatible material.

## 5 Overview of Organic Photosensitizers

The attentive reader has already noted that H_2_-producing photocatalytic systems vary from the point of view of composition, required incidence light wavelength, and overall performance. The literature describes a plethora of such systems that can be distinguished, as shown in Figure 5. The remaining part of this work is dedicated to a detailed overview of organic molecular, hybrid, and polymer-based photosensitizers.

**Figure 5:**
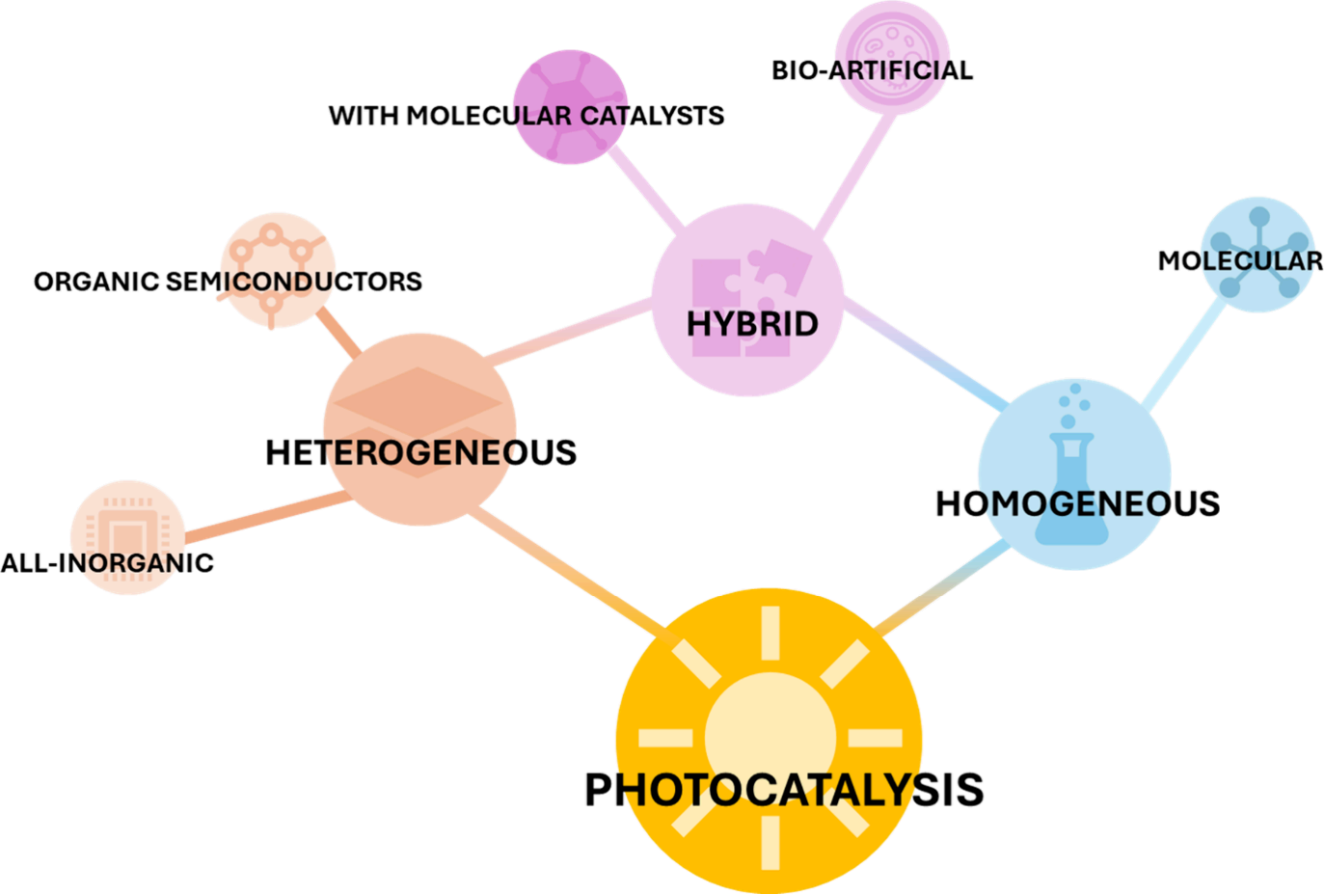
Division of light-harvesting systems for water-splitting according to the composition.

### 5.1 Molecular Organic Photosensitizers (MOPS) for HER

Molecular structures of MOPS are enriched with large delocalized π-conjugated systems, ensuring absorption of different types of electromagnetic irradiation. The ability of the PS to participate in the particular light-driven process depends essentially on the molecular construction, respectively, on the energy of the frontier molecular orbitals, determining the ground- and excited state photophysical and electrochemical attributes.

However, to be a PS, a molecule must be capable of doing more than absorbing the light, but it also has to undergo the intersystem crossing when excited to a singlet state into a relatively long-lived and energetic form of the triplet state. The importance of the triplet excited state lies in the higher probability of collision with other molecules during their longer lifetime (microseconds to seconds), resulting in the mediation of photosensitized reactions with high efficiency.^128^

A remarkable PS is characterized by its broad spectral absorption, especially in visible light, a high molar absorption coefficient, and a sufficient quantum yield of triplet state formation together with a relatively long excited-state lifetime for effective charge separation. Moreover, the value of the PS increases with the abundance of the material, the existence of short and high-yielding synthetic routes, good photostability, redox stability and chemical stability, efficient working, and competitive cost.^84,129^

In the earliest studies in the late 1970s and 1980s conducted by Lehn and Sauvage, an electron relay (electron mediator) was used, which served as an interlink for electron transfer between excited chromophores and cocatalysts.^130^ This multicomponent system was eventually simplified to systems illustrated in Figure 6 ^131^ up to supramolecular entities where photocatalysts is capable of both light absorption and H^+^ reduction.

**Figure 6:**
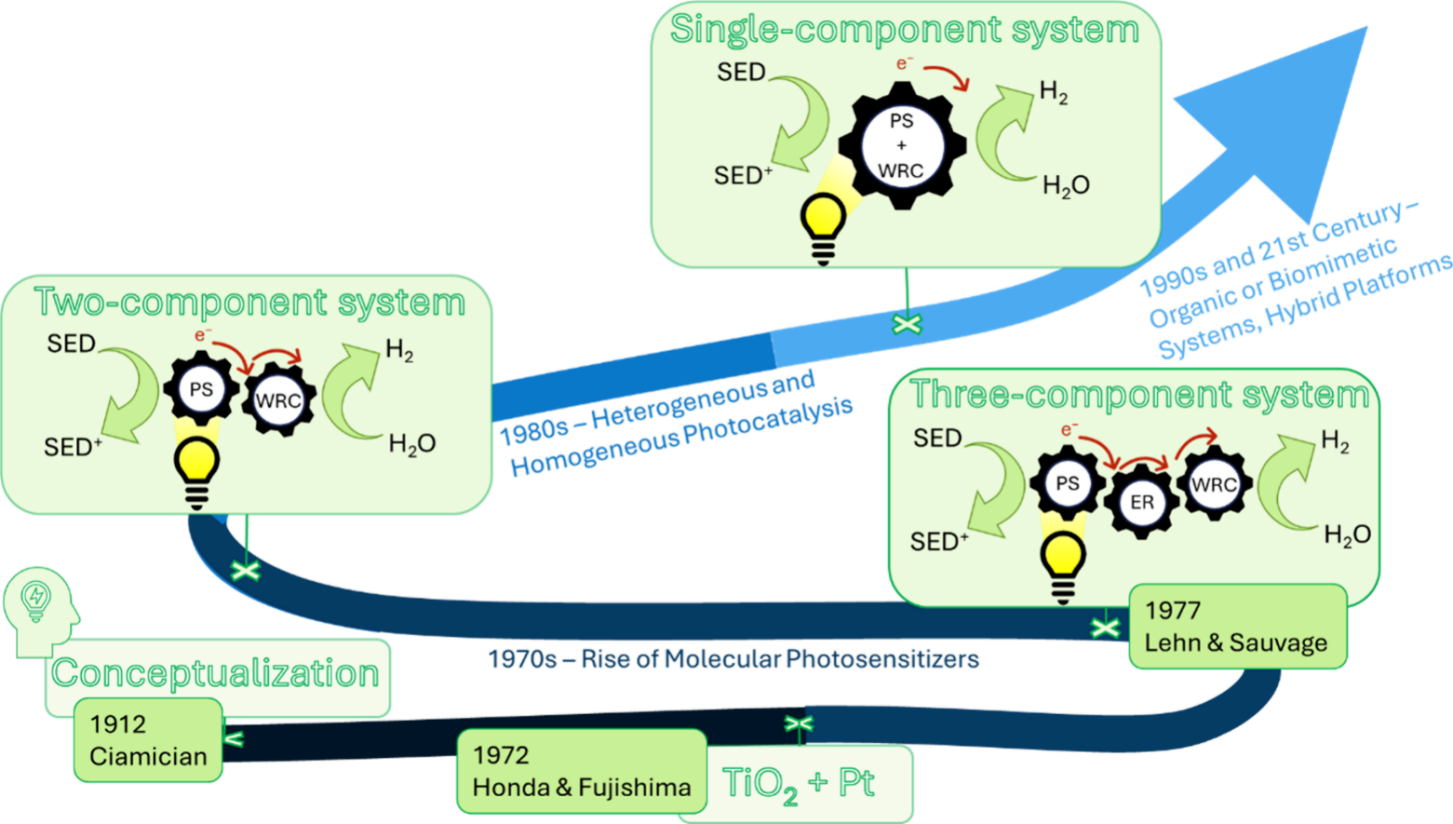
Simplified timeline of the HER systems evolution.

#### 5.1.1 Noble-Metal-Based MOPS

Noble metals are well-known for their ability to form stable coordinating complexes with ligands, resulting in compounds that exhibit remarkable optical and redox properties. These include strong visible-light absorption, long excited-state lifetime, and efficient charge transfer, ensuring a high catalytic activity, which makes them highly effective in artificial photosynthesis. Since the late 1970s, precious-metal-based organometallic complexes have played a key role in the development of systems for light-driven HER.

Historically, MOPS were first implemented in multicomponent systems involving electron mediators. In 1977, Lehn and Sauvage reported the HER using a ruthenium complex [Ru­(bpy)_3_]^2+^ (bpy = 2,2′-bipyridine) as the PS, [Rh­(bpy)_3_]^2+^ as an electron relay, colloidal platinum as WRC, and triethanolamine (TEOA) as SED.^130^ Around the same time, Kagan and co-workers^132^ and Grätzel and co-workers^133,134^ explored similar systems using methyl viologen (MV^2+^) as a metal-free redox mediator.

Although these systems were conceptually important, they were limited by the mechanistic complexity and moderate photocatalytic performance. Later developments focused on omitting electron mediators and streamlined PS-catalyst-SED systems while improving light-harvesting and redox efficiency.

The [Ru­(bpy)_3_]^2+^ complex (**1**) remains a benchmark PS due to its strong visible-light absorption (λ_max_ = 452 nm) and relatively long excited-state lifetime (τ ≈ 1.1 μs). However, its photostability is constrained by a low-lying metal-centered triplet state.^131^ Despite this, it remains widely used as a reference PS^135^ or for new cocatalyst performance assessment.^136–138^ Ligand engineering has been extensively explored to optimize redox potential, stability, and spectral properties.^139,140^ Related complexes like [Ru­(phen)_3_]^2+^ (phen = phenanthroline) (**2**), have additionally shown broader applicability in CO_2_ photoreduction^141^ (Figure 7).

**Figure 7:**
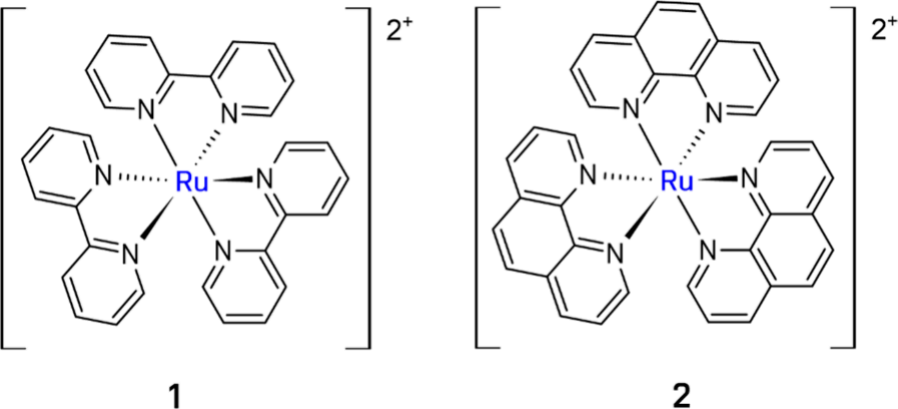
Examples of the Ru-based complexes.

Iridium­(III) complexes have emerged as particularly promising MOPS, offering stronger visible-light absorption, broader spectral coverage, and enhanced photostability due to 5d orbital participation and ligand field stabilization.^131,142^ A representative Ir­(III) complex is [Ir­(ppy)_2_(bpy)]^+^ (ppy = 2-phenylpyridine) (**3**), introduced by Goldsmith et al. in 2005, together with analogous ligands with various substituents.^143^ In combination with [Co­(bpy)_3_]^2+^ and TEOA, these complexes demonstrated HER quantum yields up to 37 × greater than [Ru­(bpy)_3_]^2+^.

Ir­(III) complexes (general formula [Ir­(ĈN)_2_(N̂N)]^+^) allow for precise electronic tuning via ligand variation (Figure 8). Typically, the HOMO is localized on the metal center and cyclometallating ĈN ligands, while the LUMO is located on the N̂N auxiliary ligand.^144^ Substitution patterns on ĈN and N̂N ligands (such as alkyl, alkoxy, halogen, or extended π-conjugation) enable systematic modulation of redox potentials and excited-state properties.^144,145^ Some complexes also incorporate structural motives that promote strong interactions with colloidal Pt cocatalysts, improving system lifetime (**4**).^146–148^

**Figure 8:**
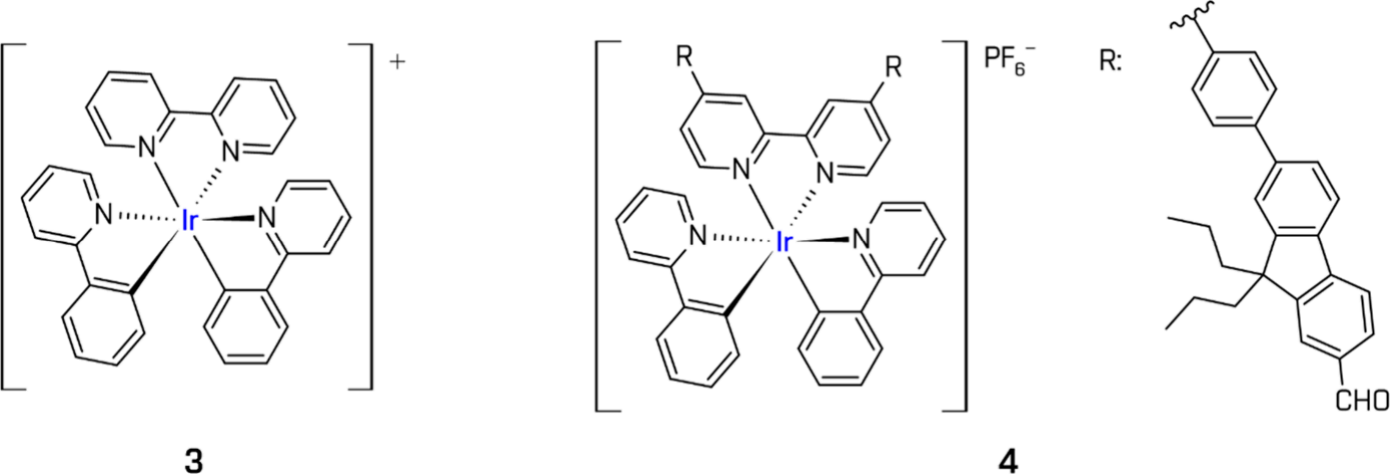
Examples of the heteroleptic Ir-based complexes. All structures are original drawings; see refs 143,147 for the corresponding literature.

Recent advances have shown that carefully tailored ligand environments around Ir­(III) and Ru­(II) centers can lead to substantial improvements in both light-harvesting efficiency and long-term stability. Wang et al. used a strategy of incorporating coumarin and BODIPY ligands, which resulted in remarkable TON and AQY when PS was coupled with [Co­(dmgH)_2_(py)­Cl] ((dmgH)_2_ = dimethylglyoxime, (py) = pyridine).^47^ Recently, Wang et al. introduced a coumarin-functionalized Ir­(III) complex incorporating triphenylamine moieties, enabling stronger visible-light absorption and improved intramolecular charge separation. The resulting system showed excellent stability under prolonged irradiation and demonstrated enhanced photocatalytic performance.^149^

In a complementary approach, Camara et al. developed a fully water-soluble sulfonated Ru­(II) complex, Na_4_[Ru­((SO_3_Ph)_2_phen)_3_] (SO_3_Ph = benzenesulfonate), with integrated sulfonic acid groups to increase hydrophilicity and facilitate operation under mild, aqueous conditions. For HER, PS paired with a cobalt-based [Co^III^(CR14)­Cl_2_]Cl cocatalyst (CR14 = 2,12-dimethyl-3,7,11,17-tetraazabicyclo[11.3.1]­heptadeca-1(17),2,11,13,15-pentaene) and ascorbate as a SED, ouperformed [Ru­(bpy)_3_]^2+^.^150^

In addition to Ru and Ir, other noble-metal-based MOPS, such as Pt­(II) and Re­(I) complexes, have also been investigated.^151,152^ A cationic Pt­(II) complex (**5**) (Figure 9) was shown to be photoactive in a multicomponent system (PS, colloidal Pt, MV^2+^, TEOA), but with lower HER efficiency compared to Ir­(III) analogues.^151^ Meanwhile, photocatalytic system ([ReBr­(CO)_3_(bpy)] (**6**), Co­(dmgH)_2_, TEOA) exhibited superior performance compared to [Ru­(bpy)_3_]^2+^ under identical light and reaction conditions.^153^

**Figure 9:**
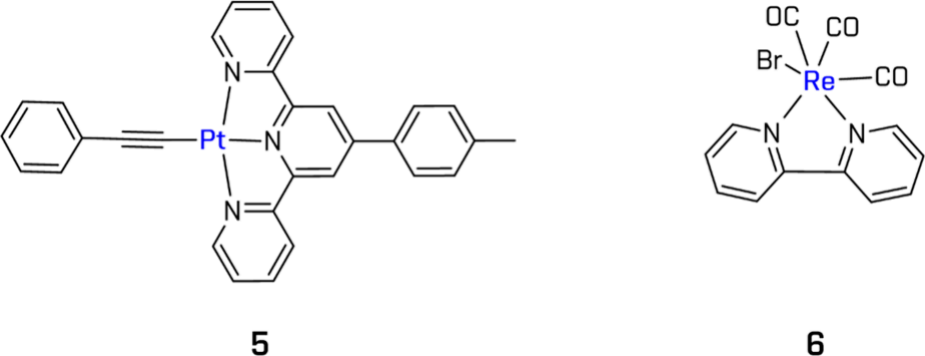
Examples of Pt- and Rebased complexes. All structures are original drawings; see refs 151,153 for the corresponding literature.

Despite their advantages, noble-metal-based MOPS face limitations in cost and scalability due to scarcity of metals like Ru, It, Pt, and Re.^131,154,155^ This has driven ongoing interest in maximizing performance and stability while minimizing metal loading.

#### 5.1.2 Noble-Metal-Free MOPS

Over the past decade, complexes of first-row transition metals have become interesting, sustainable, and cost-effective alternatives to noble-metal-based MOPS. Particular focus has been placed on zinc, copper, and iron complexes due to their earth-abundant, favorable photophysical properties, and generally lower toxicity.^84,154^

The development of new PS took inspiration from nature, where porphyrin-based pigments play key roles in light harvesting. Synthetically prepared porphyrins feature extended π-conjugation, excellent chemical stability, and strong absorption in the visible-light region via intense Soret bands (400–450 nm) and moderate Q-bands (500–600 nm).^156^ Moreover, porphyrin solubility and charge transfer properties can be tuned either vertically (via central metal chelation) or horizontally (via substituents such as sulfonate, carboxylate, or alkyl groups), supporting the design of fully aqueous and organic solvent-free HER systems.^157,158^

Early efforts by Kalyanasundaram and Grätzel introduced zinc tetra-*p*-sulfonatophenylporphyrin (ZnTPPS) (**7**) in a multicomponent HER system (ZnTPPS, colloidal Pt, MV^2+^, ethylenediaminetetraacetic acid (EDTA)) with visible light irradiation (λ > 500 nm).^159^ Later, Lazarides et al. demonstrated the feasibility of a fully noble-metal-free system using tetracationic porphyrin [ZnTMPyP]^4+^ (**8**) in combination with [Co­(dmgH)_2_(py)­Cl] and TEOA, which showed good activity and system stability^160^ (Figure 10).

**Figure 10:**
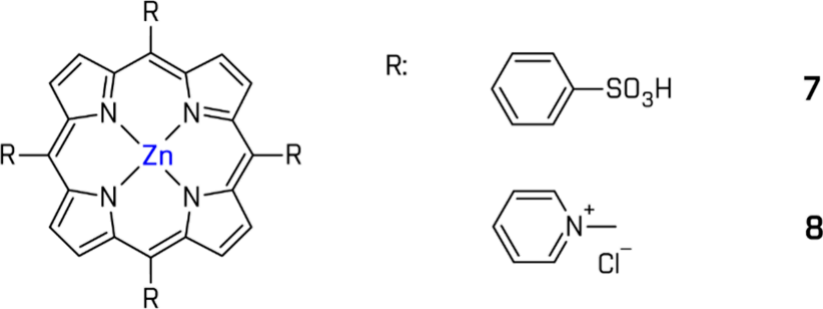
Examples of the Zn-based complexes. All structures are original drawings; see refs 159,160 for the corresponding literature.

In 2012, Luo et al. reported a series of heteroleptic Cu­(I) complexes with bathocuproine ligands that achieved HER performance exceeding TON of [Ir­(ppy)_2_(bpy)]^+^ under optimized conditions in a system with [Fe_3_(CO)_12_] and triethylamine (TEA).^161^ Moreover, homoleptic Cu­(I) complexes such as [Cu­(dsbtmp)_2_]^+^ (dsbtmp = 2,9-di­(*sec*-butyl)-3,4,7,8-tetramethyl-1,10-phenanthroline) that exhibited superior stability and enhanced visible-light absorption, particularly when paired with [Co­(dmgH)_2_(py)­Cl] and dimethyl-*p*-toluidine (DMT) as SED, were studied.^162^ Additional studies explored ligand substitution at the 2,9-positions to modulate steric and electronic effects.^163^ More recently, Chen et al. developed Cu­(II) complexes with organic dye ligands, purpurin (PP) (**9**) and gallein (GA), featuring catechol coordinating sites. These were tested in systems with Ni or Fe cocatalysts and of TEA and/or 1,3-dimethyl-2-phenylbenzimidazolin (BIH) as electron donors, demonstrating efficient visible-light-driven HER (Figure 11).^164^

**Figure 11:**
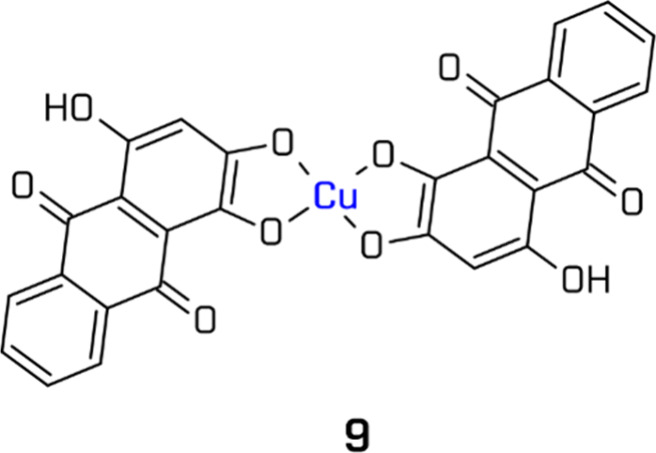
Example of the Cu-based complex. All structures are original drawings; see ref 164 for the corresponding literature.

While zinc and copper complexes have already demonstrated considerable performance for HER, other first-row transition metals such as iron and manganese are also under investigation, for their rich coordination chemistry, accessible redox states, and bioinspired relevance. Fe­(II) polypyridyl complexes are explored as analogues to classical Ru­(II) PS; however, they typically suffer from fast excited-state decay, which severely limits their ability to participate in productive photoredox processes.^154^ Manganese-based PS also offers an attractive direction; however, application in HER is hindered by short excited-state lifetimes, nonradiative decays, and susceptibility to oxidative decomposition.^154^

While the development of noble-metal-free MOPS remains active and an essential research direction, hybrid systems such as MOFs have gained increasing attention since the mid-2010s. The integration of light-harvesting and catalytic functionalities in a modular, robust framework is increasingly recognized as a next-generation direction in photocatalytic HER.^165^

#### 5.1.3 Metal-Free MOPS

In addition to metal-based complexes, several studies have explored the potential of metal-free photosensitizers. These organic compounds, primarily dyes, offer advantages such as environmental benignity, structural versatility, low cost, and the possibility of synthesis through green synthetic approaches. Their tunable optical and electronic properties, achieved through strategic molecular modifications, facilitate integration into diverse photocatalytic systems. Moreover, they demonstrate promising modularity in HER reaction systems, as they can be efficiently coupled with a wide variety of cocatalysts, enabling the creation of fully molecular HER systems.^155,166^

Despite their potential, metal-free MOPS often suffer from photodegradation, limited redox stability, fast charge recombination, or low solubility in aqueous media, all of which may reduce their catalytic lifetime or light-harvesting efficiency. Therefore, recent efforts have focused on tailoring molecular structures to enhance stability and triplet-state activity, extend light absorption ranges, and increase system robustness.^155,167,168^

Commercially available xanthene dyes such as fluorescein, Eosin Y (**10**), and rhodamines have been widely used in HER studies (Figure 12). Eosin Y, a brominated fluorescein, exhibits strong visible absorption (λ_max_ = 524 nm) and notable photosensitizing properties valuable for laboratory-scale research. So far, it has been studied in the system employing cobaloxime cocatalyst [Co­(dmgH)_2_(py)­Cl] and TEOA as SED.^169,170^ However, its broader use is limited by photodecomposition, resulting from cleavage of weak C–Br bonds under irradiation.^169,171^ Similarly, Rose Bengal (**11**), an iodine-substituted analogue, showed similar degradation behavior in multicomponent systems (Rose Bengal, colloidal Pt, MV^2+^, EDTA).^172^ To mitigate these issues, Mc. Cormick et al. introduced rhodamine-type dyes incorporating sulfur (**12**), selenium, or oxygen into the xanthene core, which improved HER activity and dye stability.^173^ Further theoretical analyses by Jiao et al. demonstrated how side-chain engineering of rhodamines could optimize their excited-state dynamics and charge-transfer efficiency.^174^

**Figure 12:**
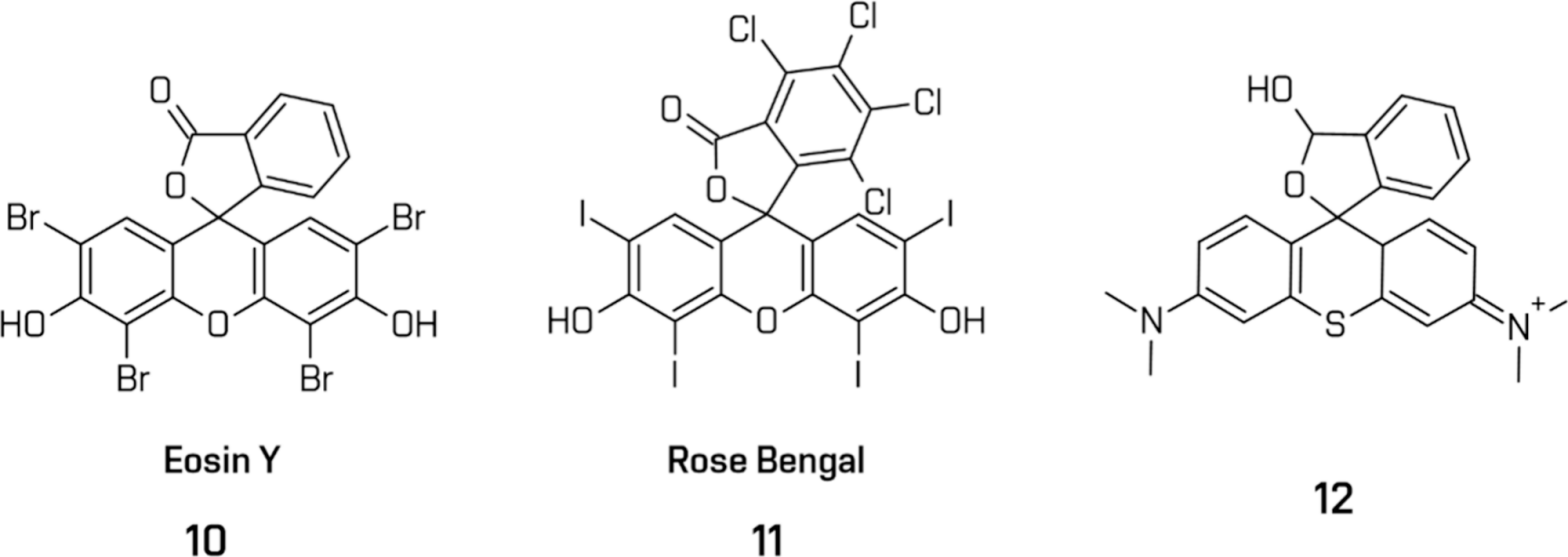
Examples of metal-free photosensitizers with xanthene core. All structures are original drawings; see refs 170,172,173 for the corresponding literature.

Boron-dipyrromethene (BODIPY) (**13**) dyes are known for their strong absorption and high fluorescence yields.^168^ However, their triplet-state population is generally low, limiting the photocatalytic performance. Introduction of heavy atoms via halogenation (Br, I) at the pyrrolic positions was introduced to increase ISC via spin–orbit coupling (**14**)^175^ (Figure 13). Bartelmess et al. developed bromo- and iodo-substituted BODIPY-cobaloxime conjugates (**15**), fulfilling the role of PS and cocatalyst for HER in the presence of TEOA.^176^ Nevertheless, photostability remains an issue, as the C-X bonds can still undergo cleavage under continuous irradiation.^177^ Chemical modifications and linker strategies are being applied to mitigate these pathways and extend dye lifetimes.^176,178^

**Figure 13:**
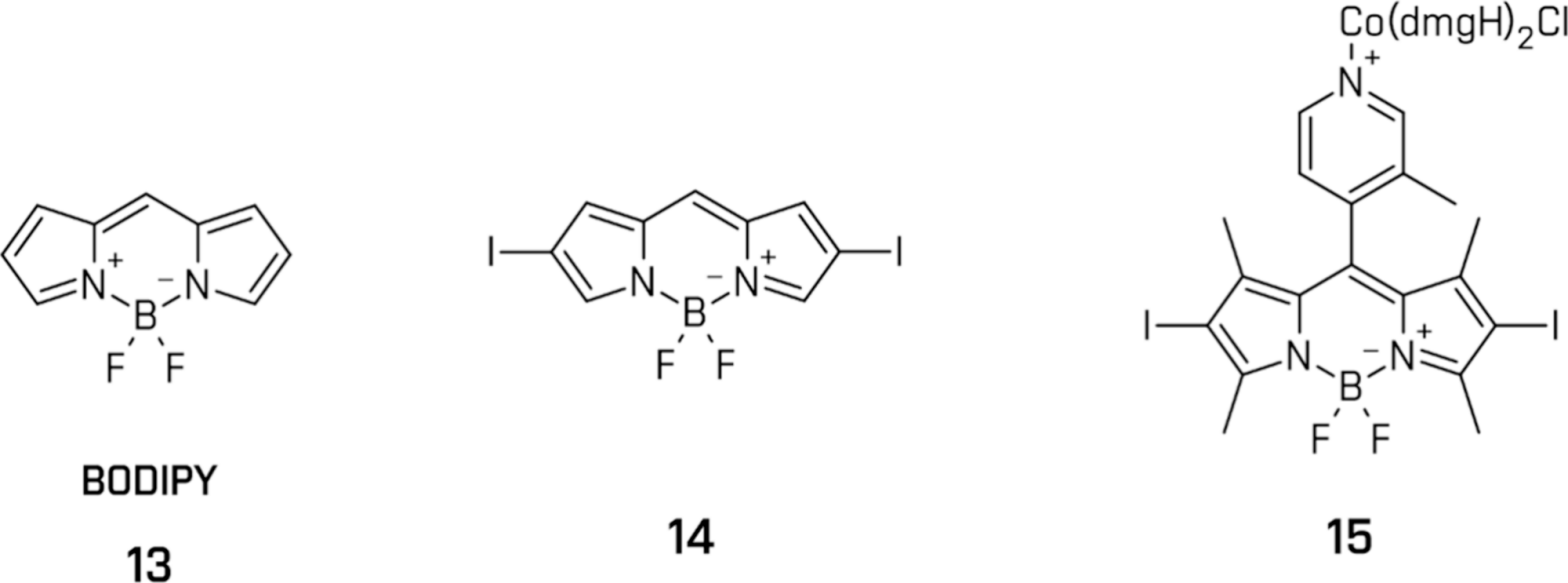
Examples of the BODIPY dyes and the cobaloxime complex. All structures are original drawings; see refs 175,176 for the corresponding literature.

Several purely organic photosensitizers, composed only of C, H, O, and N, have been explored (Figure 14). Early systems (1978) using proflavine (**16**) demonstrated that simple aromatic molecules could drive HER, albeit at modest efficiencies.^179^ Decades later, acriflavine (**17**), a commercially available cationic dye, showed notably higher HER rates when paired with cobaloxime compared to colloidal Pt under similar conditions.^180^

**Figure 14:**
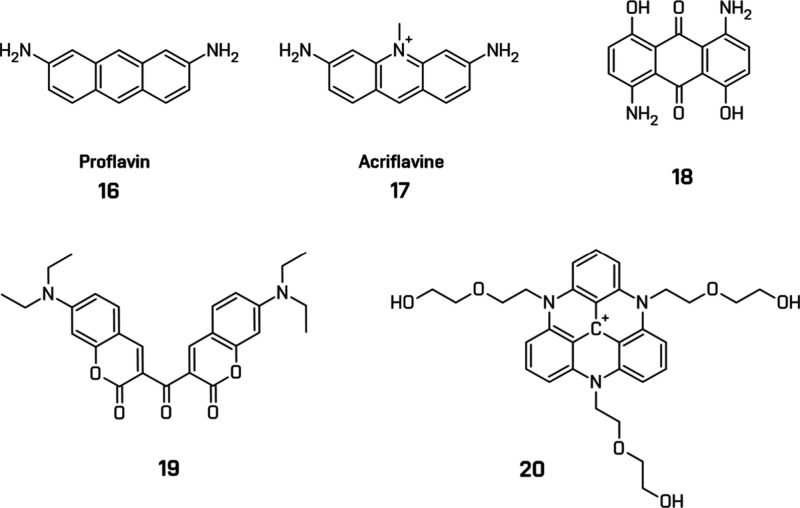
Examples of the heavy-atom-free metal-free photosensitizers. All structures are original drawings; see refs 135,179–181,183 for the corresponding literature.

Coumarins are another well-studied class, valued for their availability and photostability, but often limited by low quantum yield of ISC. Dong et al. demonstrated a 61 × increase in activity by using keto-dicoumarin dimers (**19**),^181^ and Knorr et al. reported high TONs with ketocoumarine derivatives coupled with [Co­(dmgH)_2_(py)­Cl] and sodium ascorbate in water under both UV and visible-light irradiation. Their work remains one of the highest HER efficiencies for cobaloxime-organic dye systems.^182^

Among recent innovations in metal-free MOPS, triazatriangulenium (TATA^+^) derivatives stand out due to their exceptional photostability, structural rigidity, and strong visible-light absorption. Gueret et al. introduced water-soluble TATA^+^ functionalized with ethoxyethanol side groups (**20**). The resulting system, paired with a cobalt tetraazamacrocyclic catalyst [Co^III^(CR14)­Cl_2_]^+^, and ascorbate, outperformed benchmark [Ru­(bpy)_3_]^2+^ systems in fully aqueous media under visible-light irradiation.^135^

While most organic PS operate in the broad-to-green region of the visible spectrum, this range accounts for only about 20% of the solar irradiance reaching the Earth’s surface. To utilize a broader portion of sunlight, recent efforts have focused on red- and near-infrared (NIR)-absorbing dyes, including anthraquinone derivatives.

A notable example is 1,5-diamino-4,8-dihydroxyanthraquinone (DAHA) (**18**) introduced by Ming et al. with strong, red-shifted absorption (λ_max_ = 614 nm) extending to the NIR region. Their implementation in HER systems facilitates efficient red-light-driven hydrogen evolution with a significantly high AQY.^183^

Flavin-based photosensitizers have recently emerged as promising metal-free candidates due to their modular structure, visible-light absorption, and synthetic flexibility. Ivanová et al. reported that alkylated alloxazine derivatives, while not water-soluble, can function efficiently in aqueous media when combined with an appropriate cosolvent and cocatalyst. These systems demonstrated improved photostability and performance under mild, low-intensity irradiation, reinforcing flavins as a sustainable platform for further optimization.^184^

In summary, significant progress has been achieved across all classes of MOPS, including noble-metal-based, noble-metal-free, and fully metal-free systems. Advancements in molecular design, photostability, and spectral tuning have contributed to improved performance in photocatalytic hydrogen evolution. Nevertheless, several key limitations remain, such as poor solubility in aqueous environments, short excited-state lifetimes, and susceptibility to photodegradation under prolonged irradiation. Moreover, performance comparison is often complicated by the variability in system architectures and testing conditions. The problem with comparing the performance of photosensitizers across different studies is further complicated by unclear standards on reporting the photophysical and photocatalytic properties. Still, Table 3 summarizes the photocatalytic efficiency of selected photosystems.

**Table 3:** Overview of Some of the Photocatalytic Systems with Reported TON, TOF and/or AQY Values

photosensitizer	cocatalyst	sacrificial reagent	TON	TOF/h^–1^	AQY	ref.
[Ru(bpy)_3_]Cl_2_	Co-phthalocyanine	TEA	2400	680	4.2% @420 nm	^138^
coumarin-Ir-BODIPY	[Co(dmgH)_2_(py)Cl]	DMT	115,840		37.7% @475 nm25.1% @520 nm	^47^
[Ir(coumarin)_2_(TPAbpy)][PF_6_]	K_2_PtCl_4_	ascorbic acid	198,363	3209	0.07% @450 nm	^149^
[ReBr(CO)_3_(bpy)]	Co(dmgH)_2_	TEOA	150		26% @415 nm	^153^
Na_4_[Ru((SO_3_Ph)_2_phen)_3_]	[Co^III^(CR14)Cl_2_]Cl	sodium ascorbate, ascorbic acid	4772	1095		^150^
[ZnTMPyP]Cl_4_	[Co(dmgH)_2_(py)Cl]	TEOA	280			^160^
Cu-purpurin	(Et_4_N)-Ni-(5-H-pyS)_3_pyS = pyridine-2-thiolate	BIH, TEA	1180	9.8	0.14% @450 nm	^164^
[Cu(bathocuproine)(DPEphos)][PF_6_]	[Fe_3_(CO)_12_]	TEA	477			^161^
diprotonated dodecaphenylporphyrinH_4_DPP^2+^	poly(vinylpyrrolidone)-protected Pt nanoparticles	10-methyl-9,10-dihydroacridine			17% @710 nm12% @480 nm8% @750 nm	^185^
Eosin Y	[Co(dmgH)_2_(py)Cl]	TEOA	900		4% @520 nm	^169^
Se-substituted Rhodamine	[Co(dmgH)_2_(py)Cl]	TEOA	9000	5500	32.8% @520 nm	^173^
7-(diethylamino)-3-(3,4,5-trimethoxybenzoyl)-2H-chromen-2-one	[Co(dmgH)_2_(py)Cl]	sodium ascorbate	3821		0.26% (UV lamp)	^182^
tris(ethoxyethanol)triazatriangulenium TATA^+^	[Co^III^(CR14)Cl_2_]^+^	sodium ascorbate, ascorbic acid	8952	6500		^135^
DAHA	[Co(dmgH)_2_(py)Cl]	BIH, NH_4_Br	787,000	7033	30.6% @630 nm	^183^
10,12-dibutylpyreno[4,5-g]pteridine-11,13(10*H*,12*H*)-dione	K_2_PtCl_4_	TEA	400			^184^

As an alternative approach, increasing attention has been directed toward polymeric and hybrid organic systems, which offer enhanced structural robustness and the integration of multiple functional components within a single material platform and will be discussed in the following chapters.

### 5.2 Hybrid and Polymeric Organic PS Platforms

Recent advances in molecular photosensitizers have increasingly extended beyond isolated small molecules toward structured and hybrid materials. These systems aim to address key limitations of classical MOPs—namely, limited photostability, inefficient charge separation, and poor long-term recyclability—by embedding photosensitizing units within functional matrices or polymeric frameworks. More broadly, hybrid architectures have attracted considerable attention in artificial photosynthesis because they can combine the tunability of molecular components with the robustness, interfacial control, and functional integration typical of heterogeneous platforms, including systems relevant to coupled redox transformations.^186^ Platforms such as metal–organic frameworks (MOFs), conducting polymers (CPs), and polydopamine (PDA) offer new opportunities to enhance light-harvesting efficiency, tune charge transfer dynamics, and improve structural robustness under photocatalytic conditions, as schematically illustrated in Figure 15. In this chapter, we examine representative examples of these emerging materials, highlight their performance in hydrogen evolution systems, and discuss their potential to bridge the gap between molecular tunability and material scalability.

**Figure 15:**
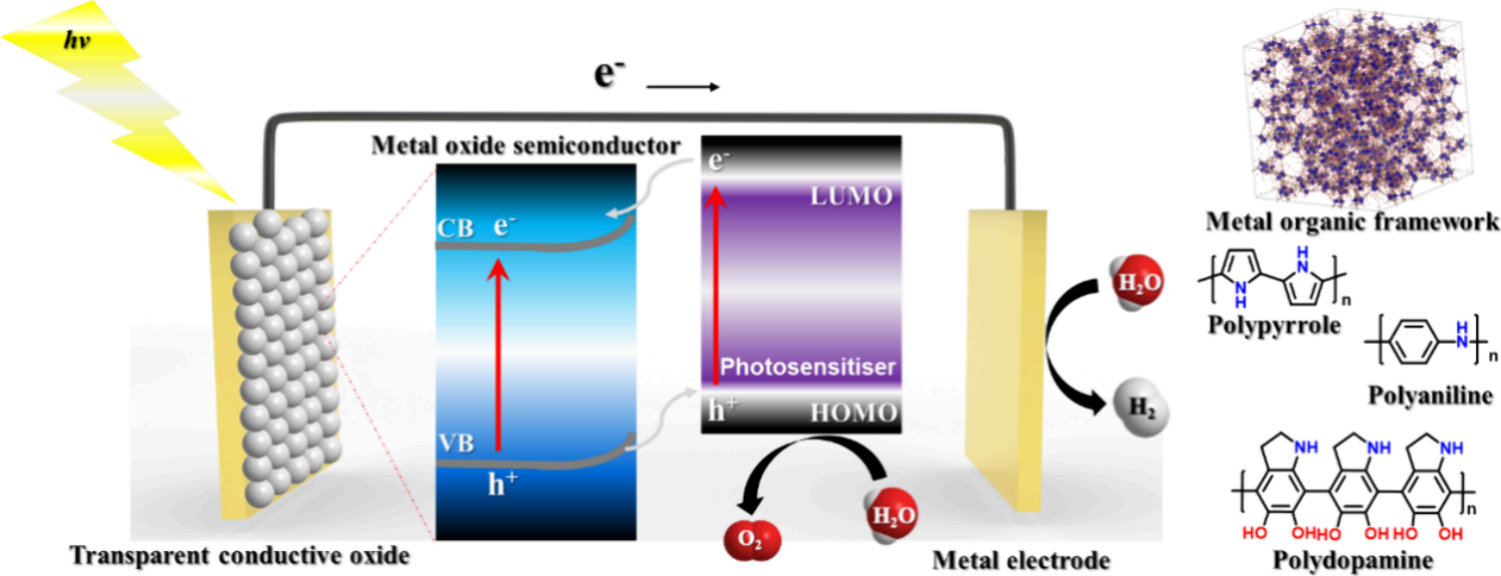
Schematic of the role of photosensitizers in hydrogen evolution, highlighting energy transfer and catalytic pathways, with molecular structures of MOFs, CPs, and PDA.

#### 5.2.1 Metal Organic Frameworks (MOFs)

Metal–organic frameworks are crystalline materials formed by the coordination of metal ions or clusters with organic ligands, resulting in porous, scaffold-like structures that mimic biological functions. Their hierarchical design, encompassing multiple levels of organization, allows for precise control over composition and structure through modular synthesis strategies, with key scientific disciplines such as coordination chemistry and nanoscience contributing to MOF development.^187–191^ With tunable structures and dynamic properties, MOFs offer unique advantages over traditional inorganic materials, including their potential to minimize interpenetration—a phenomenon where multiple frameworks interlock—through strategic design. Their photoactive organic linkers impart semiconductor-like characteristics, enabling effective light absorption and electron transfer, which are crucial for applications such as photocatalytic water splitting. The structural versatility of MOFs enables modifications with various organic linkers and metal ions, enhancing their physicochemical properties and promising improved performance in energy storage as precursors for stable porous metal oxide nanostructures, which can significantly enhance supercapacitor performance.^192,193^ MOFs also play a vital role in enhancing PEC devices by improving aspects of photoelectrodes, such as efficient carrier separation, charge injection, and light absorption range, optimizing solar-to-chemical energy conversion.^194^ Liu et al. reported that the MIL-101­(Fe)/Mo photoanode, with a MOF layer, increases photocurrent density and efficiency by extending visible light response and providing accessible active sites for water oxidation, leading to improved stability in photoelectrochemical processes.^195^ Similarly, Dong et al. demonstrated that the NH_2_-MIL-101­(Fe) MOF also acts as an effective PS in Fe_2_O_3_/Fe-based core/shell nanorods for PEC water oxidation, where its ultrathin shell significantly enhances visible light absorption and charge separation, improving hole injection and catalytic efficiency for oxygen evolution.^196^ Tang et al. explored how MOF-derived Co_3_C nanosheets improve the PEC performance of TiO_2_ hollow cages by forming type-II heterojunctions, which promote electron–hole separation, accelerate surface water oxidation, and extend light harvesting, contributing to increased photocurrent density and efficiency.^197^ Zhang et al. highlighted that MOFs, with highly ordered structures and large surface areas, also enable periodic arrangements of light-harvesting and catalytic components akin to natural photosynthesis, showcasing their potential for solar energy conversion.

In particular, Ti-based MOFs show promising photocatalytic properties by photogenerating electrons in organic ligands and efficiently transferring them to Ti-oxo clusters, significantly improving the PEC water oxidation performance of TiO_2_ under visible light.^198^ Liu et al. reported the development of a novel p-type nickel-based MOF single crystal (Ni-TBAPy-SC) and its exfoliated nanobelts (Ni-TBAPy-NB) with exceptional water stability across a broad pH range, paving the way for visible-light-driven photocatalytic water splitting. Their study demonstrates efficient electron transfer from the H_4_TBAPy ligand, acting as the light-harvesting center, to the Ni–O cluster node, which serves as the catalytic center, enabling hydrogen production without the need for a cocatalyst. The exfoliated nanobelts exhibited enhanced charge separation and greater active surface areas compared to those of the single crystal, owing to their reduced charge transfer distance. These improvements resulted in a remarkable 164-fold increase in the water reduction activity. Notably, the nanobelts achieved an optimal hydrogen evolution rate of 98 μmol h^–1^ (approximately 5 mmol h^–1^ g^–1^), with a benchmark AQE of 8.0% at 420 nm, making them among the most effective water-stable MOF photocatalysts to date.^113^ Their unique ability to integrate both light-harvesting and catalytic functionalities within a periodic, porous matrix underscores their potential in solar-driven applications. This capability offers a promising perspective for future use as a PS, particularly in applications where enhanced light capture, carrier mobility, and chemical stability are paramount.

#### 5.2.2 Conducting Polymers (CPs)

Conducting polymers are utilized as PS in PEC applications to enhance visible light absorption and facilitate charge transfer to inorganic semiconductors. When combined with materials like TiO_2_ or ZnO, CPs extend the spectral range and reduce electron–hole recombination, leading to improved PEC performance.^199^ Polymers such as polyaniline (PANI) exhibit strong light absorption in the visible range, which enhances the photocatalytic activity. Additionally, the positively charged backbone of PANI promotes interactions with negatively charged dyes, improving adsorption and degradation efficiency, while PANI composites effectively suppress electron–hole recombination to enhance charge separation and catalytic performance. PANI-modified systems, such as CCTO and PdSeCdS composites, also demonstrate enhanced hydrogen evolution and corrosion resistance under visible light. When combined with renewable materials like cellulose aerogel, PANI provides eco-friendly, high-surface-area supports that prevent catalyst agglomeration, supporting sustainable catalytic applications.^200–202^ Bu et al. reported that the PANI/Ag/Ag_3_PO_4_ composite, with 20 wt % PANI, shows significantly enhanced photocatalytic activity for Rhodamine B degradation—about four times higher than pure Ag_3_PO_4_. In this way, this improvement is due to the formation of a heterojunction electric field (∼90 mV) between PANI and Ag_3_PO_4_, which enhances electron–hole pair separation and accelerates hole transfer, preventing Ag_3_PO_4_ self-oxidation.^203^ In another application, Thanh Truc et al. utilized PANI and polythiophene (PTh) to sensitize tantalum nitride (Ta_3_N_5_), enhancing its photocatalytic activity for water splitting under visible light. These polymers serve as charge acceptors by facilitating hole migration and protecting Ta_3_N_5_ from oxidation and photocorrosion. Notably, Ta_3_N_5_/PANI demonstrates superior hydrogen and oxygen production rates because PANI has higher electrical conductivity than PTh.^204^ L. Liu et al. further explored the Ag_3_PO_4_@PANI core–shell photocatalyst, prepared via chemisorption, and achieved enhanced photocatalytic performance with increased PANI content, leading to 100 and 95.3% degradation of phenol and 2,4-dichlorophenol, respectively. This catalyst shows high stability (85%) after five cycles, outperforming other Ag_3_PO_4_-based systems because the π-conjugated structure of PANI enhances charge separation and prevents the dissolution of Ag_3_PO_4_ during reactions.^205^ Thanh Truc et al. demonstrated that niobium-doped Ta_3_N_5_ sensitized with polypyrrole (NbeTa_3_N_5_/PPy) exhibits high photocatalytic efficiency for water splitting under visible light by enhancing electron–hole separation and charge transfer. Moreover, the niobium doping introduces an intermediate band, improving charge carrier separation, while PPy supports electron and hole migration, reducing recombination, and protecting Ta_3_N_5_ from photocorrosion. This catalyst demonstrates excellent stability, achieving H_2_ and O_2_ production rates of 65.1 and 32.8 mmol g^–1^ h^–1^, respectively.^206^ Luo et al. reported that polypyrrole-TiO_2_ nanotube arrays (PPy-TNTs) synthesized via anodization and potentiostatic polymerization exhibit a tunable PPy content that enhances visible-light sensitivity. Therefore, the p–n junctions between PPy and TNTs improve charge separation and transfer, resulting in a photocurrent density 2.5 times higher than that of pristine TNTs under simulated solar light, along with enhanced photoelectrochemical stability and increased photon-to-current conversion efficiency in both UV and visible light regions.^207^ From a future perspective, conducting polymers are poised to become highly adaptable and efficient PS in PEC applications due to their tunable light absorption and charge transport properties. Their compatibility with various inorganic semiconductors and renewable materials enables the creation of eco-friendly, high-performance hybrid systems tailored for sustainable energy solutions. As research continues to optimize CPs for improved stability, light harvesting, and charge separation, they hold significant potential to drive advances in solar energy conversion and environmental remediation.

#### 5.2.3 Polydopamine (PDA)

Polydopamine is a bioinspired polymer developed based on the adhesive proteins found in mussels and was first introduced in 2007. In a weakly alkaline solution, dopamine undergoes oxidation and self-polymerization to form a thin film that strongly adheres to a wide range of surfaces, facilitated by functional groups such as catechol and amine that enable metal ion binding and robust adhesion to various materials.^208–210^ The biocompatibility and environmentally friendly properties of PDA make it versatile for applications in energy storage, environmental remediation, and biosensing.^210^ For instance, nitrogen and sulfur dual-doped carbon nanotubes synthesized using PDA as a precursor achieved uniform and high sulfur doping, resulting in bifunctional electrocatalytic performance for both the hydrogen and oxygen evolution reactions (HER and OER) in alkaline solutions, surpassing other carbon-based catalysts due to improved charge transfer and the creation of highly active catalytic sites from dual doping.^211^ Gao et al. reported that PDA also enabled the confined growth and uniform dispersion of a Co–Pi cocatalyst on BiVO_4_, forming the Co–Pi/PDA/BiVO_4_ heterostructure. This structure significantly enhanced PEC performance with a photocurrent density of 2.47 mA cm^–2^ at 1.23 V vs RHE and improved charge injection efficiency from 25 to 75%.^212^ Ruan et al. integrated PDA with CdS to form a stable inorganic–organic hybrid heterostructure that enhanced electron transfer and improved photoelectrode stability. As a result, a photocurrent density of 1.08 mA cm^–2^ was achieved, 2.4 times higher than that of bare CdS. Furthermore, the introduction of a Co–Pi cocatalyst further boosted the water oxidation efficiency, reaching a photocurrent density of 2.68 mA cm^–2^, 5.7 times that of bare CdS.^213^ G. Ryu et al. reported that a hematite-based PEC biosensor platform developed for NADH detection utilized PDA-coated hematite, which immobilized a redox mediator (NB), thus enhancing electron transfer and NADH oxidation. This approach achieved a detection limit of 4.65 μM and sensitivity of 12.8 mA mM^–1^, demonstrating reliable glucose sensing in plasma.^214^ Polydopamine stands out as a valuable material for a wide range of applications due to its exceptional adhesive properties, compatibility with biological systems, and robust chemical stability. Its functional groups enable strong interfacial bonds with different substrates, while its versatile nature supports the development of hybrid structures that enhance the charge transfer and catalytic durability. With the potential to further expand applications in biomedicine, energy, and environmental science, PDA presents a promising future perspective for enhancing sustainable and versatile materials across fields.

## 6 Conclusion and Outlook

Organic photosensitizers have evolved from early model systems to a broad and diverse class of functional materials for light-driven hydrogen evolution. Synthetic accessibility and compatibility with earth-abundant elements allow for careful modulation of redox and photophysical properties. Recent efforts have expanded the field beyond discrete molecules toward polymeric and hybrid platforms that improve structural stability, charge mobility, and recyclability. While many challenges remain—particularly in achieving long-term stability, reproducibility, and performance in water-based environments—recent progress has demonstrated that well-designed organic compounds can compete with conventional materials in both activity and selectivity.

Future research is expected to benefit from interdisciplinary strategies combining molecular photochemistry, materials engineering, and device-level integration. Key directions include the design of heavy-atom-free and fully metal-free OPS with tailored absorption profiles, the rational incorporation of photosensitizers into robust frameworks, and the development of testing protocols that allow for meaningful comparison across systems. Continued progress in these areas will be essential for advancing from academic investigations toward practical applications in green hydrogen technologies.

## Supplementary Material



## Notes

Discussion of the sacrificial electron donors and water reduction catalysts (PDF)
