# Supplementary material for: A Review on Organic Photosensitizers for Hydrogen Evolution by Water Splitting
Source: ACS Omega. 2026 Apr 13;11(16):23670–90. doi: 10.1021/acsomega.6c01074 (PMC13129843; doi:10.1021/acsomega.6c01074)
Supplement: Supplementary file 1 [file ao6c01074_si_001.pdf]

A REVIEW ON ORGANIC PHOTOSENSITISERS FOR HYDROGEN EVOLUTION BY WATER SPLITTING

Supporting Information

Lucia Ivanová [a)], Jan Truksa [a)*], Kyusun Kim [b), c)], Dong Ryeol Whang [d)], Bong Sup Shim [c) †], Jozef Krajčovič[a)†]*

[a)]Faculty of Chemistry, Brno University of Technology, Purkyňova 118, CZ-612 00 Brno, Czech Republic

[b)]Center for Clean Technology, Inha University, 100, Inha-ro, Michuhol-gu, Incheon 22212, Republic of Korea

[c)]Department of Chemical Engineering & Program in Biomedical Science and Engineering, Inha University, 100, Inha-ro, Michuhol-gu, Incheon 22212, Republic of Korea

[d)] Department of Advanced Materials, Hannam University, Daejeon, 34054, Republic of Korea

[†]corresponding author:

krajcovic@fch.vut.cz (J. Krajčovič)

phone: +420541149433

bshim@inha.ac.kr (B. Shim)

phone: +82328607477

[*]Equally contributed

Sacrificial electron donors

The presence of an SED **alters the thermodynamics of the reaction**, possibly making the overall process more energetically favourable by lowering the required free energy. Therefore, in many cases, it is no longer valid to talk about artificial photosynthesis when considering these systems. Further, it is necessary to take care when using metrics tied to a specific Gibbs energy, such as η_{STH}, which may also be invalidated. Additionally, SED can **enhance reaction kinetics** by promoting charge separation and reducing electron-hole recombination. Ideally, after passing the electron to PS, irreversible monoelectronic oxidation must occur to degrade SED into inert molecules that will not interfere with the desired pathways of the photocatalytic system.

The selection of an SED depends on its compatibility with the PS, ensuring favourable redox potentials for efficient electron transfer. Additionally, the degradation of the oxidized donor (SED^{+}) must be faster than its recombination with the reduced PS (PS^{-}) [1]. Other key factors include solubility in the reaction medium and stability under photocatalytic conditions. Commonly used tertiary aliphatic amines include triethylamine (TEA) and triethanolamine (TEOA). These are effective in pure organic solvents, organic-water mixtures (e.g., *N*,*N*-dimethylformamide, tetrahydrofuran, acetonitrile), or, in the case of TEOA, directly in aqueous media. Their degradation pathways are well-documented [2]. In acidic conditions, ethylenediaminetetraacetic acid (EDTA) is often employed as a two-electron donor in aqueous solutions, as protonated amines lose their electron-donating ability [1]. Other reported SEDs include aromatic amines (e.g., *N*,*N*-dimethyl-p-toluidine (DMT), 1,3-dimethyl-2-phenyl-2,3-dihydro-1H-benzo[d]imidazole (BIH)), alcohols (methanol, ethanol), thiols, and ascorbic acid [1,3]. Some of these structures are illustrated in **Figure S1**.

While traditional sacrificial reagents are often costly and mainly suited for laboratory use, research is shifting toward greener alternatives for practical applications. This shift aims to improve both efficiency and sustainability. Promising electron donors include glucose, biomass-derived methanol, and glycerol. Additionally, readily available biomass components like cellulose, hemicellulose, lignin, and even plastic waste are being explored as viable options [4].

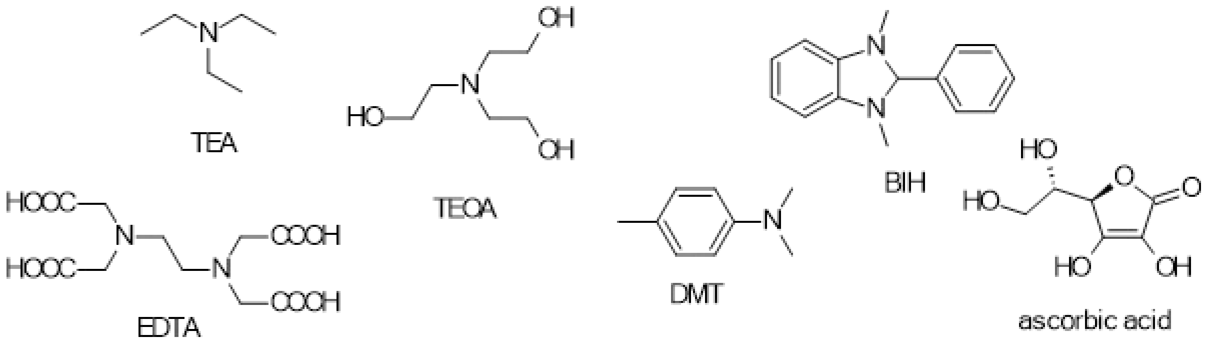

Figure S1 Typically used electron donors used for reductive quenching in HER.

Water reduction catalysts

Water-reduction catalysts (WRC), often referred to as co-catalysts, are essential components of photocatalytic HER. They facilitate the transfer of electrons from the excited PS to protons and provide redox-active sites necessary for H_2 generation. Noble metals, particularly colloidal platinum (Pt) have been widely employed due to their high efficiency and electron-trapping ability [5–9]. These nanoparticles exhibit large surface area and have served as benchmark catalysts in molecular HER systems. However, their non-molecular nature can influence reaction kinetics, stability, and mechanistic interpretation, and such systems may be more appropriately classified as “hybrid” or “quasi-molecular”.

True molecular WRC, based on metal complexes with well-defined coordination

So far, many true molecular co-catalysts have been reported, and the progress is often reviewed [5,10–14]. The metal ions in these systems are typically coordinated with various ligands, including, e.g., bi- and polypyridines, porphyrins, tetraene-N_4 macrocycles, η^5-cyclopentadienides, phosphines, dimethylglyoximes, their derivatives.

As alternatives to colloidal Pt, researchers are investigating platinum-based WRCs [15] along with other noble-metal-based WRCs [16,17]. Despite their effectiveness, the scarcity and high cost of precious metals prevent their large-scale application in H_2 production. Consequently, there is a consensus within the scientific community for developing more affordable and readily available alternatives [18,19].

Recent research has highlighted the potential of first-row transition metals and earth-abundant materials as viable co-catalysts for HER. Typically, metals of the "iron triad" – Fe, Co, and Ni are known for their catalytic properties and are considered viable substitutions. For instance, Gärtner et al. proposed iron carbonyl WRCs within the system using $[Ir(ppy)_2(bpy)]^+$ as PS and TEA as SED [20]. In addition, Na et al. demonstrated H_2 production using model diiron complexes resembling hydrogenase enzymes utilized by nature, combined with the photosensitiser $[Ru(bpy)_3]^{2+}$ and ascorbic acid as SED [10,21].

Cobalt-based co-catalysts appear among the most promising candidates for effective and stable molecular WRCs. Especially, complexes with oxime ligands (e.g., called $[Co(dmgH)_2(py)Cl]^{2+}$, where $(dmgH)_2$ = dimethylglyoxime, (py) = pyridine) have demonstrated activity in the tandem with either (noble-)metal-based PSs or metal-free organic dye PSs. The latter studies present an approach to reaching efficient hydrogen production with solely earth-abundant elements [22].

References

1. Pellegrin Y, Odobel F. Sacrificial electron donor reagents for solar fuel production. *Comptes Rendus Chimie*. 2016;20(3):283-295. doi:10.1016/j.crci.2015.11.026
2. Stoll T, Castillo CE, Kayanuma M, et al. Photo-induced redox catalysis for proton reduction to hydrogen with homogeneous molecular systems using rhodium-based catalysts. *Coord Chem Rev*. 2015;304-305:20-37. doi:10.1016/j.ccr.2015.02.002
3. Schneider J, Bahnemann DW. Undesired Role of Sacrificial Reagents in Photocatalysis. *J Phys Chem Lett*. 2013;4(20):3479-3483. doi:10.1021/jz4018199
4. Zhang T, Lu S. Sacrificial agents for photocatalytic hydrogen production: Effects, cost, and development. *Chem Catalysis*. 2022;2(7):1502-1505. doi:10.1016/j.checat.2022.06.023
5. Artero V, Fontecave M. Solar fuels generation and molecular systems: is it homogeneous or heterogeneous catalysis? *Chem Soc Rev*. 2013;42(6):2338-2356. doi:10.1039/C2CS35334B
6. Lehn JM, Sauvage JP. Chemical storage of light energy-catalytic generation of hydrogen by visible-light or sunlight-irradiation of neutral aqueous-solutions. *Nouv J Chim*. 1977;1:449-351.
7. Kiwi J, Grätzel M. Hydrogen evolution from water induced by visible light mediated by redox catalysis. *Nature*. 1979;281(5733):657-658. doi:10.1038/281657a0
8. Kalyanasundaram K, Kiwi J, Grätzel M. Hydrogen Evolution from Water by Visible Light, a Homogeneous Three Component Test System for Redox

Catalysis. *Helv Chim Acta*. 1978;61(7):2720-2730. doi:10.1002/hlca.19780610740

9. Keller P, Moradpour A, Amouyal E, Zidler B. Sacrificial hydrogen generations from water mediated by a series of viologen-dye relays. *Journal of Molecular Catalysis*. 1981;12(2):261-263. doi:10.1016/0304-5102(81)80013-2

10. Mazzeo A, Santalla S, Gaviglio C, Doctorovich F, Pellegrino J. Recent progress in homogeneous light-driven hydrogen evolution using first-row transition metal catalysts. *Inorganica Chim Acta*. 2021;517:119950. doi:10.1016/j.ica.2020.119950

11. Gueret R, Poulard L, Oshinowo M, et al. Challenging the $[Ru(bpy)_3]^{2+}$ Photosensitizer with a Triazatriangulenium Robust Organic Dye for Visible-Light-Driven Hydrogen Production in Water. *ACS Catal*. 2018;8(5):3792-3802. doi:10.1021/acscatal.7b04000

12. Probst B, Guttentag M, Rodenberg A, Hamm P, Alberto R. Photocatalytic H_2 Production from Water with Rhenium and Cobalt Complexes. *Inorg Chem*. 2011;50(8):3404-3412. doi:10.1021/ic102317u

13. Bartelmess J, Francis AJ, El Roz KA, Castellano FN, Weare WW, Sommer RD. Light-Driven Hydrogen Evolution by BODIPY-Sensitized Cobaloxime Catalysts. *Inorg Chem*. 2014;53(9):4527-4534. doi:10.1021/ic500218q

14. Lazarides T, Delor M, Sazanovich I V., et al. Photocatalytic hydrogen production from a noble metal free system based on a water soluble porphyrin derivative and a cobaloxime catalyst. *Chem Commun*. 2014;50(5):521-523. doi:10.1039/C3CC45025B

15. Sakai K, Ozawa H. Homogeneous catalysis of platinum(II) complexes in photochemical hydrogen production from water. *Coord Chem Rev*. 2007;251(21-24):2753-2766. doi:10.1016/j.ccr.2007.08.014

16. Elvington M, Brown J, Arachchige SM, Brewer KJ. Photocatalytic Hydrogen Production from Water Employing A Ru, Rh, Ru Molecular Device for Photoinitiated Electron Collection. *J Am Chem Soc*. 2007;129(35):10644-10645. doi:10.1021/ja073123t

17. Cline ED, Adamson SE, Bernhard S. Homogeneous Catalytic System for Photoinduced Hydrogen Production Utilizing Iridium and Rhodium Complexes. *Inorg Chem*. 2008;47(22):10378-10388. doi:10.1021/ic800988b

18. Corredor J, Rivero MJ, Rangel CM, Gloaguen F, Ortiz I. Comprehensive review and future perspectives on the photocatalytic hydrogen production. *Journal of Chemical Technology & Biotechnology*. 2019;94(10):3049-3063. doi:10.1002/jctb.6123

19. Xiao N, Li S, Li X, Ge L, Gao Y, Li N. The roles and mechanism of cocatalysts in photocatalytic water splitting to produce hydrogen. *Chinese Journal of Catalysis*. 2020;41(4):642-671. doi:10.1016/S1872-2067(19)63469-8

20. Gärtner F, Sundararaju B, Surkus A, et al. Light-Driven Hydrogen Generation: Efficient Iron-Based Water Reduction Catalysts. *Angewandte Chemie International Edition*. 2009;48(52):9962-9965. doi:10.1002/anie.200905115

21. Na Y, Wang M, Pan J, Zhang P, Åkermark B, Sun L. Visible Light-Driven Electron Transfer and Hydrogen Generation Catalyzed by Bioinspired [2Fe2S] Complexes. *Inorg Chem*. 2008;47(7):2805-2810. doi:10.1021/ic702010w

22. Lazarides T, McCormick T, Du P, Luo G, Lindley B, Eisenberg R. Making Hydrogen from Water Using a Homogeneous System Without Noble Metals. *J Am Chem Soc*. 2009;131(26):9192-9194. doi:10.1021/ja903044n
